# Extracellular vesicles released by human retinal pigment epithelium mediate increased polarised secretion of drusen proteins in response to AMD stressors

**DOI:** 10.1002/jev2.12165

**Published:** 2021-11-08

**Authors:** Miguel Flores‐Bellver, Jason Mighty, Silvia Aparicio‐Domingo, Kang V. Li, Cui Shi, Jing Zhou, Hannah Cobb, Patrick McGrath, German Michelis, Patricia Lenhart, Ganna Bilousova, Søren Heissel, Michael J. Rudy, Christina Coughlan, Andrew E. Goodspeed, S. Patricia Becerra, Stephen Redenti, M. Valeria Canto‐Soler

**Affiliations:** ^1^ *CellSight* Ocular Stem Cell and Regeneration Program Department of Ophthalmology Sue Anschutz‐Rodgers Eye Center University of Colorado, School of Medicine Aurora Colorado USA; ^2^ Lehman College Bronx New York USA; ^3^ Biology Doctoral Program The Graduate School and University Center City University of New York New York New York USA; ^4^ Department of Dermatology University of Colorado School of Medicine Aurora Colorado USA; ^5^ Section of Protein Structure and Function NEI NIH Bethesda Maryland USA; ^6^ Charles C. Gates Center for Regenerative Medicine University of Colorado School of Medicine Aurora Colorado USA; ^7^ Linda Crnic Institute for Down Syndrome University of Colorado School of Medicine Aurora Colorado USA; ^8^ Proteomics Resource Center The Rockefeller University New York New York USA; ^9^ University of Colorado Alzheimer's and Cognition Center Department of Neurology Linda Crnic Institute for Down Syndrome University of Colorado Anschutz Medical Campus Aurora Colorado USA; ^10^ Department of Pharmacology University of Colorado Anschutz Medical Campus Aurora Colorado USA; ^11^ University of Colorado Cancer Center University of Colorado Anschutz Medical Campus Aurora Colorado USA; ^12^ Biochemistry Doctoral Program The Graduate School City University of New York New York New York USA; ^13^ Department of Neurology University of Colorado School of Medicine Aurora Colorado USA

**Keywords:** AMD, drusen, exosomes, extracellular vesicles, microvesicles, proteomics, RPE, stem cells

## Abstract

Age‐related macular degeneration (AMD) is a leading cause of blindness worldwide. Drusen are key contributors to the etiology of AMD and the ability to modulate drusen biogenesis could lead to therapeutic strategies to slow or halt AMD progression. The mechanisms underlying drusen biogenesis, however, remain mostly unknown. Here we demonstrate that under homeostatic conditions extracellular vesicles (EVs) secreted by retinal pigment epithelium (RPE) cells are enriched in proteins associated with mechanisms involved in AMD pathophysiology, including oxidative stress, immune response, inflammation, complement system and drusen composition. Furthermore, we provide first evidence that drusen‐associated proteins are released as cargo of extracellular vesicles secreted by RPE cells in a polarised apical:basal mode. Notably, drusen‐associated proteins exhibited distinctive directional secretion modes in homeostatic conditions and, differential modulation of this directional secretion in response to AMD stressors. These observations underpin the existence of a finely‐tuned mechanism regulating directional apical:basal sorting and secretion of drusen‐associated proteins via EVs, and its modulation in response to mechanisms involved in AMD pathophysiology. Collectively, our results strongly support an active role of RPE‐derived EVs as a key source of drusen proteins and important contributors to drusen development and growth.

## 1 INTRODUCTION

A hallmark of ageing in the eye is the appearance of drusen, extracellular deposits that form between the basal lamina of the RPE and the inner collagenous layer of the Bruch's membrane (Green, 1999). The presence of numerous drusen in the macula is considered a major risk factor for the development of advanced age‐related macular degeneration (AMD) (Ambati et al., 2003), a leading cause of blindness and visual impairment, affecting millions of individuals worldwide (Wong et al., 2014). Advanced dry AMD is characterised by focal atrophy of the RPE and loss of macular photoreceptors, whereas choroidal neovascularisation, also known as wet AMD, involves abnormal blood vessel growth from the choriocapillaris through the RPE (Bhutto & Lutty, 2012). Although drusen are widely accepted as contributors to the etiology of both dry and wet AMD, little is known about its biogenesis. A better understanding of this process could lead to therapeutic strategies to modulate drusen biogenesis, and in turn, to slow or halt the progression of AMD.

Emerging evidence suggests that extracellular vesicles (EVs), such as exosomes, microvesicles and exomeres, may participate in the pathogenesis of AMD (Klingeborn et al., 2017; Lakkaraju et al., 2020). EVs have been defined as a double‐edged sword since they can both, promote disease progression or support homeostasis maintenance (Baixauli et al., 2014; Xu et al., 2018). Importantly, while the mechanisms of propagation of RPE dysfunction in AMD remain a critical gap in our understanding of the disease, EVs play a role in spreading the toxic forms of aggregated proteins in other neurodegenerative diseases (Alvarez‐Erviti et al., 2011; Rajendran et al., 2006). It has been observed that EV cargo reflects the nature and physiology of their cell of origin, and any change in cell homeostasis might modify the molecular composition of EVs (Baixauli et al., 2014; Colombo et al., 2014). Thus, the cargo contained in RPE‐derived EVs during AMD may reflect the type and physiological–pathological state of the RPE cells. Notably, to date, the characterisation of the proteome cargo of EVs from human RPE tissue in homeostatic conditions, and analysis of changes induced by an AMD‐like environment have not been accomplished.

Human induced pluripotent stem cells (hiPSCs) (Takahashi & Yamanaka, 2006) provide unprecedented opportunities for the development of human cell‐based models to study diseases (Canto‐Soler et al., 2016). Here, we demonstrate that the RPE tissue present in our hiPSC‐derived retinal organoids is analogous to the native human RPE and establish a method to derive functionally mature polarised RPE monolayers analogous to human primary RPE. Furthermore, we demonstrate the ability of our RPE monolayers to recapitulate key features of AMD, including drusen‐like deposits, when exposed to chronic oxidative stress, and use this system to analyse the dynamics of EV release and protein cargo in homeostatic and AMD‐like environments. We demonstrate that under homeostatic conditions, RPE‐derived EVs are selectively enriched in proteins involved in oxidative stress, immune response, inflammation, complement system and drusen composition. Furthermore, we provide the first evidence that drusen‐associated proteins are released as cargo of extracellular vesicles from RPE cells through a directional sorting mechanism, which is modulated in response to AMD‐linked stressors.

## 2 METHODS

### 2.1 Generation of induced‐primary RPE (ipRPE) from human retinal organoids: Cell culture and reagents

The use of human iPSCs in this study conforms to the University of Colorado Office of Regulatory Compliance. A human induced pluripotent stem cell (hiPSC) line derived from CD34+ cord blood was used for all experiments in this study (A18945, ThermoFisher Scientific) (Burridge et al., 2011). Two additional cell lines were used to confirm reproducibility of ipRPE culture: a human primary neonatal fibroblast‐derived line (IN2–5) (Kogut et al., 2018); and a human primary dermal fibroblast derived (ic4‐4) (Kogut et al., 2018). All cell lines were obtained with verified normal karyotype and contamination‐free. Cell culture, retinal differentiation, and human retinal organoid (hRetOs) formation were conducted as previously described by our group (Zhong et al., 2014), including best practices standardised techniques consistent with Zhong et al. (2014) and Capowski et al. (2019). Briefly, hiPSC were maintained on Matrigel (growth‐factor‐reduced; BD Biosciences) coated plates and their pluripotency state was confirmed by expression of pluripotency markers OCT4, NANOG and SSEA4. After 6 days in culture, hiPSC colonies were lifted and cultured as free‐floating neural aggregates (NAs); this was established as Day 0 (D0) of differentiation. On D7, NAs were seeded onto Matrigel (growth‐factor‐reduced; BD Biosciences) coated dishes, and individual mechanical detachment of the NR and RPE domains was perform on D14. Isolation and culture of RPE cells from hRetOs was performed as follows: RPE spheroids were dissected from hRetOs and dissociated into small aggregates by incubating for 4 h in DMEM supplemented with 0.25% (wt/vol) collagenase type IV (17104019, ThermoFisher Scientific) at 37°C followed by mechanical dissociation by vigorous pipetting (glass Pasteur pipette, 50x). At that point, RPE aggregates were enzymatically dissociated into single cells by another incubation for 30 min with Accumax (07921, Stem Cells Technology) followed by gentle mechanical dissociation (P1000 micropipette, 10×). The RPE single cell solution was filtered through a 40‐μm nylon mesh (352340, BD Falcon) to discard non‐dissociated aggregates. Next, RPE single cells were centrifuged, resuspended, and seeded at 150,000 cells/cm^2^ onto Transwell filters (Corning Costar, 3460‐Clear, 0.4 mm pores, 12 mm inner diameter, polyester membranes, Fisher Scientific, cat. no. 07‐200‐161) coated with Matrigel (growth‐factor‐reduced; BD Biosciences). ipRPE monolayers were cultured at 37°C and 5% CO_2_ in alpha MEM media (M‐4526, Sigma) containing, 1 × N1 supplement (N‐6530, Sigma), Taurine (T‐0625, Sigma), Triiodo‐thyronin (M‐4526, Sigma), Hidrocortisone (T‐5516, Sigma), non‐essential amino acids (M‐7145, Sigma) and Glutamine‐penicillin‐streptomycin (G‐1146, Sigma), and 2%–5% of FBS‐depleted exosomes (EXO‐FBSHI‐250A‐1, System Biosciences, SBI) following the cell culture media formulation established by Maminishkis et al. (2006) for primary human fetal RPE cultures with minor modifications. These culture conditions are referred as homeostatic conditions (Klingeborn et al., 2017). Cell culture media was changed every other day by adding 0.5 ml of cell media to the upper compartment and 1.5 ml to the lower compartment. ipRPE monolayers were passaged every 10 days after incubation with Accumax for 30 min. Undifferentiated hiPSCs, hRetOs and ipRPE were routinely tested for Mycoplasma contamination by PCR.

### 2.2 Cigarette smoke extract (CSE) treatment

Cigarette smoke extract, containing 40 mg/ml condensate and 6% nicotine, was purchased from Murty Pharmaceuticals (Lexington, KY), and was prepared by smoking University of Kentucky's 3R4F Standard Research Cigarettes on an FTC Smoke Machine. The smoke on the filter is calculated by the weight gain of the filter after smoking. The condensate is extracted with DMSO by soaking and sonication, then packaged 1 ml/vial in dry vials. CSE was prepared fresh daily for single use, sterile filtered using 0.22 μm Millex syringe filters (EMD Millipore, Billerica, MA, USA) and diluted to the desired concentration in serum‐free RPE cell culture media. For acute oxidative stress conditions ipRPE monolayers were treated with 0, 50, 100 and 200 μg/ml CSE in DMSO for 24 h. For chronic oxidative conditions, ipRPE monolayers were treated with 0 and 100 μg/ml CSE in DMSO every other day for 4 weeks.

### 2.3 Immunofluorescence

Human retinal organoids were fixed in 4% paraformaldehyde for 1 h, washed in PBS (2 × 5 min), and cryoprotected with a sucrose gradient (6.75%, 12.5% and 25%, overnight at 4°C each) with a final incubation in 25% sucrose/OCT (1:1 ratio respectively) for 1 h at room temperature. Samples were embedded in 25% sucrose/OCT Tissue‐Tek (Sakura), frozen, and stored at –80°C until use. Cryosections of 12–16 μm thickness were obtained and collected on Superfrost Plus slides. Sections were air dried for 1 h and washed in PBS (3 × 5 min). ipRPE monolayers were fixed in 4% paraformaldehyde for 10 min, washed in PBS (3 × 5 min) and stored at 4°C until processed. All samples were blocked in 10% goat serum in PBS with 0.25% Triton X‐100 for 1 h at RT, and incubated overnight with a primary antibody in 2% goat serum in PBS with 0.05% Triton X‐100 at 4°C. The next day, samples were washed in PBS (3 × 5 min) and incubated with an Alexa Fluor‐conjugated secondary antibody (1:500; Molecular Probes) in PBS for 2 h in the dark at RT. The samples were then washed in PBS (3 × 5 min), incubated in DAPI (1:1000 in PBS) for 10 min, and cover‐slipped using DAKO fluorescent mounting medium. Similar procedures were applied for double immunostaining. Fluorescence images were acquired with a Nikon C2 laser scanning confocal microscope (Melville, NY, USA). The images were minimally processed using Adobe Photoshop CS5 (San Diego, CA, USA). Image J software (National Institutes of Health, Bethesda, MD, USA) was used to prepare movies. Information for all antibodies used, including company, catalog number and concentration, is provided in Table S6.

### 2.4 Western blot

ipRPE monolayers were scraped in ice‐cold PBS and lysed with 200 μl RIPA buffer (Sigma‐Aldrich, St. Louis, MO, USA) and Protease Inhibitor Cocktail (Sigma–Aldrich). Proteins from EVs released by ipRPE monolayers were extracted using 300 μl of RIPA buffer and protease inhibitor cocktail. All samples were vortexed six cycles for 30 s, kept in ice for 15 min and stored at –20°C until further processing. Protein content was determined by microBCA assay (ThermoFisher Scientific, catalog # 23235). The samples were loaded in a 10% NuPAge Bis‐Tris Plus gel (NW0302BOX, Invitrogen). Electrophoresis was performed at constant 120 V for 2 h. The proteins in the gel were transferred to a nitrocellulose membrane using iBlot (Invitrogen). The membrane was then blocked with 5% BSA in TBST for 1 h and incubated with a primary antibody overnight at 4°C. The next day, membranes were washed three times with TBST for at least 10 min, followed by incubation for 2 h at room temperature in horseradish peroxidase‐conjugated antibodies. Bands were visualised with ECL (Pierce, Thermo Scientific, Rockford, IL, USA) and detected with Image Quant LAS‐4000 mini (GE Healthcare, Uppsala, Sweden). The results were normalised using beta (β)‐tubulin protein as a loading control (detected with E7 antibody from Developmental Studies Hybridoma Bank). Protein levels were quantified by densitometry from three biological independent samples using ImageJ software (National Institutes of Health, Bethesda, MD, USA); signal intensity was normalised to that of b‐actin. Information for all antibodies used is provided in Table S6.

### 2.5 Transepithelial resistance (TER)

TER measurements of ipRPE monolayers cultured on Transwell filters were performed using an epithelial volt‐ohm meter (EVOM, World Precision Instruments, Sarasota, FL) according to Sonoda et al.(2009). Briefly, the electrodes of the EVOM were sterilised with 70% ethanol, rinsed in Hank's balanced salt solution, and placed in the Transwell. The tip of the longer electrode was immersed into the basolateral medium (lower chamber) and shorter electrode into the apical medium (upper chamber). All TER measurements were performed within 3 min of removal of Transwells from the incubator since TER fluctuates with temperature. Net TERs were calculated by subtracting the value of a blank, Matrigel‐coated Transwell without cells from the experimental value. Final resistance‐area products (Ω x cm^2^) were obtained by multiplying by the effective growth area.

### 2.6 Enzyme‐linked immunosorbent assay of VEGF and PEDF secretion

VEGF and PEDF proteins were measured in media samples collected from the ipRPE monolayer apical and basal sides (upper and lower compartments of the Transwell, respectively) 48 h after complete media change on days 15, 30 and 50 of differentiation. VEGF and PEDF levels were measured with commercially available ELISA kits (VEGF: DVE00; R&D Systems; PEDF: PED613; XpressBio), following the manufacturer's instructions.

### 2.7 Reverse transcription‐polymerase chain reaction and quantitative real‐time polymerase chain reaction

ipRPE monolayers were incubated in RNA Protect (Qiagen, Hilden, Germany) to attenuate endogenous RNAse activity and mRNA synthesis and scraped off the plate into a 1.5‐ml tube. Cells were centrifuged at 2500 × *g* for 10 min and the pellet was resuspended in buffer RLT plus (RNeasy Plus Micro/Mini Kits; Qiagen) with 2‐mercaptoethanol (1:100; Sigma–Aldrich Corp.). RNA extraction was performed according to manufacturer's protocol (RNeasy Micro/Mini Kits; Qiagen). Reverse transcription polymerase chain reactions (RT‐PCR) were performed with SuperScript III First‐Strand Synthesis System (Life Technologies, Thermo Fisher Scientific) at either 30 or 35 cycles, and subsequent PCR products were run on 2% agarose gels. For quantitative real‐time PCRs (qRT‐PCR), reactions were performed with PerfeCTa Sybr Green SuperMix (Quantabio) and a Q qPCR instrument (Quantabio). All qRT‐PCR reactions were run at 40 cycles. Quantitative PCR samples were run in triplicate and, in all cases, expression levels were normalised using three housekeeping genes: HRPT1, SRP72 and HMBS. The geometric mean of the reference genes was used to standardise the results. Information for all primers used is provided in Table S7.

### 2.8 Phagocytosis assay

Phagocytosis assays were performed on ipRPE monolayers on transwells at D50 of differentiation. Photoreceptor outer segments (POS) were isolated as previously described by Schertler and Hargrave (2000) from freshly obtained cow eyes (J.W. Treuth & Sons). POS pellets were stored at –80°C until use. Different techniques were used to evaluate phagocytosis: detection of beta‐hydroxybutyrate production (Bullock et al., 2021); western blot analysis to detect rhodopsin (Bullock et al., 2021); and confocal imaging to detect POS internalisation. First, ipRPE cells were incubated with unlabelled bovine POS (1 × 10^7^ POS/ml) for 5 h in ringers‐carnitine working solution and 5 mM glucose and the apical media was collected and stored (–80°C) until use for beta‐hydroxybutyrate detection. Subsequently, the ipRPE monolayers were washed with PBS 10 times by vigorous pipetting to remove any POS that had not been phagocytosed and ipRPE monolayers were either fixed and immunostained for confocal imaging or processed for Western Blot analysis.

### 2.9 Detection of beta‐hydroxybutyrate production

Release of beta‐hydroxybutyrate by ipRPE monolayers was measured with and without exposure to bovine POS (1 × 10^7^ POS/ml for 5 h) (Bullock et al., 2021). Cell conditioned media was filtered, centrifuged and stored until use at –80°C. Beta‐hydroxybutyrate was detected with a commercially available kit (The Stanbio B‐Hydroxybutyrate Liquicolor kit (SBHR100); Fisher Scientific) following manufacturer's guidelines.

### 2.10 Transmission electron microscopy (TEM)

ipRPE monolayers were fixed in cold 2.5% glutaraldehyde/2% PFA phosphate buffer, in 1% osmium tetroxide, dehydrated and embedded in Eponate. 50 nm ultra‐thin sections were cut and stained with uranyl acetate and lead citrate. Preparations were imaged using transmission electron microscopy (Hitachi H7600). EVs released by ipRPE monolayers were isolated using differential centrifugation. Five microlitres of suspension containing isolated EVs was dropped on a Zoo‐mesh Carbon Formuar grid for 30 min at room temperature. Excess suspension was wicked off and grids were submerged in 25% glutaraldehyde/4% PFA with 25% tannic acid in PBS for 10 min. Grids were then washed in distilled water and viewed on a FeiTecnai transmission electron microscope, operated at 60kv. Digital images were obtained using an AMT digital camera and software.

### 2.11 Isolation of induced‐primary RPE‐derived EVs

EVs were isolated following guidelines and standards from MISEV2018 (Théry et al., 2018). We have submitted all relevant data of our experiments to the EV‐TRACK knowledgebase (EV‐TRACK ID: EV210262) (Van Deun et al., 2017). EVs were isolated from conditioned culture media from ipRPE cultured in transwells for 48 h. A total of 0.5 ml was collected from the apical side and 1.5 ml from the basal side per each well (12 well plates). Media was collected from transwell plates (12 inserts per plate) with a total of 8,400,000 million ipRPE cells per plate. Media was centrifuged at 300  ×  *g* for 10 min at 4°C to pellet cell debris and stored at –80°C. Briefly, supernatant was transferred to an ultracentrifuge tube (Beckman Coulter) and spun at 10,000  ×  *g* for 40 min using 70Ti.1 rotor (Beckman Coulter ultracentrifuge); supernatant was filtered through 0.22 μm filter and centrifuged at 100, 000  ×  *g* for 90 min to pellet the EVs. All centrifugations were performed at 4°C to minimise degradation of EVs. Isolated apical‐EVs and basal‐EVs from different experimental conditions were resuspended in PBS and stored at −80°C for Western Blot analysis. In addition, two independent EV samples containing apical and basal secreted EVs from homeostatic or CSE conditions were pooled and analyzed by Western Blot and NanoSight analysis.

### 2.12 Iodixanol density gradient separation

EVs apically released by ipRPE were separated by floatation into an iodixanol density gradient. Briefly, OptiPrepTM (Sigma D1556) solutions (40%, 20%, 10% and 5%) were consecutively layered into a 13.2 ml centrifuge tube (Beckman Coulter Cat 344059). After EV‐enriched fractions were concentrated by ultracentrifugation (as described in section 2.11), 1 ml of the resuspended pellet was added to the top of the density gradient and centrifuged at 141,000 × *g* for 60 h at 4°C in a Beckman Coulter L8‐55 M ultracentrifuge with a SW‐41 swinging bucket rotor. When centrifugation was completed, consecutive aliquots were collected from the top of the density gradient and labelled ‘Fraction 1‘ for the least‐dense, top‐most‐aliquot all the way down to the most‐dense ‘Fraction 10‘ at the bottom of the tube. The refractive index for each fraction was experimentally determined. Finally, the fractions were diluted in PBS and centrifuged at 100,000 × *g* for 90 min in a 70Ti.1 rotor. Pellets were resuspended in lysis solution and protein content was determined by microBCA assay (ThermoFisher Scientific, catalog # 23235).

### 2.13 NanoSight analysis

EV size and concentration were assessed using the NanoSight NS500 system. Based on the NanoSight protocol, to ensure accurate readings, final supernatant was diluted at 1:20 in PBS and triplicates of 1 ml samples were used for analysis. The NanoSight system uses a laser light source to illuminate nano‐scale particles, detected individually as light‐scattered points moving via Brownian motion. Polydispersity was quantified, and Nanoparticle Tracking Analysis (NTA) software 2.3 used to track size and diffusion of nanoparticles. Results are displayed as a frequency size distribution graph, describing the number of particles per millilitre.

### 2.14 Determination of ROS levels

Levels of reactive oxygen species (ROS) were measured using dihydroethidium (DHE; ThermoFisher Scientific). DHE is oxidised by superoxide to form 2‐hydroxyethidium and by non‐specific oxidation by other sources of ROS to form ethidium. ipRPE monolayers were rinsed twice with PBS and incubated with cDMEM containing 5 uM of DHE for 30 min at 37°C. After rinsing the cells once in cDMEM, intracellular ROS production was measured by a fluorescence multiplate reader (TECAN Infinite M1000). Fluorescence scanning parameters used for excitation: wavelength/bandwidth, 498/5 nm; and emission wavelength/bandwidth, 590/10 nm.

### 2.15 TUNEL assay

Fixed ipRPE monolayers were stained using in situ cell death detection kit conjugated with tetra‐methyl‐rhodamine or fluorescein isothiocyanate (Roche) according to the manufacturer's instructions. For controls, terminal deoxynucleotidyl transferase enzyme was either omitted from the labelling solution (negative control), or an ipRPE monolayer was incubated with 1000 U/ml DNase I recombinant for 10 min at RT to induce DNA strand breaks, prior to labelling procedures (positive control). Fluorescence images were acquired with a Nikon C2 laser scanning confocal microscope (Melville, NY, USA).

### 2.16 Mass spectrometry

Proteins were precipitated with ice‐cold acetone and pellets were dissolved in 8 M urea, 50 mM ammonium bicarbonate. Disulfide bonds were reduced using dithiothreitol and alkylated using iodoacetamide. Proteins were digested using lysyl endopeptidase (Wako) and sequencing grade trypsin (Promega) and peptides were purified using in‐house constructed STAGE‐tips. Peptides were separated using a pulled‐emitter C18 column (75 μm*120 mm, 3 μm material, Nikkyo Technos, Japan) across a 122‐min linear gradient going from 6%–38% solvent B (80% acetonitrile, 0.1% formic acid in water) in solvent A (0.1% formic acid in water). Gradient was delivered at 200nl/minute using a Dionex 3000 HPLC (Thermo Scientific). The mass spectrometer (Q‐Exactive Plus, Thermo Scientific) operated in positive DDA mode picking the 20 most abundant precursors for HCD fragmentation each duty cycle.

### 2.17 Proteomic profiling analysis

Mass spectrometry data was analysed using MaxQuant v. 1.6.6.0. Spectra were queried against the human proteome (downloaded from uniprot.org (Feb 2019), 73931 sequences) using a 1% FDR on both PSM, peptide and protein level. Oxidation of M and acetylation of protein N‐terminals were included as variable modifications and carbamidomethylation of C was included as static modification. Matching between runs was enabled, and further data analysis was performed using Perseus v. 1.6.5.0. Four EV samples were used for further analysis. Intensity‐Based Absolute Quantitation (iBAQ) values were log(2x) transformed for further data analysis. EV‐specific proteins were identified as those that were identified in at least two of the EV replicates. Pathway enrichment was performed using the clusterProfiler R package Yu et al. (2012) with gene sets from the MSigDB (Liberzon et al., 2011). Selected pathways were plotted. In addition, the bioinformatics network mapping software FunRich v. 3.1.4 Pathan et al. (2015) was utilised to analyse EV protein molecular function, biological processes and molecular pathways. The current version of ExoCarta hosts 41,860 proteins, of which 5549 are human proteins (Keerthikumar et al., 2016).

### 2.18 Coculture of ipRPE‐EGFP derived EVs and mRNA uptake assay

A transgenic hiPSC line expressing EGFP previously described and validated for differentiation of hRetOs by our group Vergara et al. (2017) was used. This transgenic EGFP‐hiPSC line was generated by electroporation with the Neon Transfection System (Invitrogen) according to manufacturer instructions as described in Ranganathan et al. (2014). The following plasmid DNAs were used: CRISPR/Cas9‐mediated constitutively expressed GFP hiPSC line: 1 μg of AAV‐CAGGS‐EGFP donor vector (Addgene # 22212, gift from Rudolf Jaenisch (Hockemeyer et al., 2009)); 0.6 μg of pCas9_GFP (Addgene # 44719, gift from Kiran Musunuru); and 0.3 μg of gRNA_AAVS1‐T2 (Addgene # 41818, gift from George Church (Mali et al., 2013)). Briefly, hiPSCs were pre‐treated with 5 μM blebbistatin for 24 h to increase cell viability, followed by treatment with Accutase (Stemcell Technologies) for 5 min, dissociated into single cells, centrifugated at 80 × *g* for 5 min to pellet the cells and incubated on ice for 15 min. The corresponding plasmids were combined in R buffer, resuspended in the plasmid cocktail and electroporated with a 10 μl tip‐type and the following parameters: 1300 V; 20 ms pulse length; 1 pulse. Cells were then gently resuspended into 1 ml of mTeSR1 plus 5 μM blebbistatin, incubated at room temperature for 20 min and plated onto Matrigel‐coated 35 mm TC treated dishes containing mTeSR1 and 5 μM blebbistatin. Finally, cells were incubated at room temperature for 20 min and cultured thereafter in 37°C and 5% CO_2_. After 5 days, stable clonal sublines were manually selected with a Leica MZ‐16F fluorescence stereomicroscope. EGFP‐ipRPE monolayers cultured in transwells were generated using this EGFP‐hiPSC line and EVs were isolated from conditioned media by differential centrifugation. Apical EVs derived from EGFP‐ipRPE at day 50 of differentiation were cocultured with non‐fluorescent (wt) ipRPE monolayers during 48 h by adding 5 × 10^6^ EVs per transwell. After incubation, wt ipRPE monolayers were rinsed twice with PBS and cells were collected for RNA extraction to detect EGFP‐mRNA by RT‐PCR and qPCR analysis.

### 2.19 Coculture of protein‐labelled ipRPE‐derived EVs and protein uptake assay

For EV protein uptake studies and, according to the manufacturer's instructions, 20 ul of 10^11^ EVs/ml were resuspended in 500 μl of PBS and 1 μl of 500X ExoGlow‐Protein EV Labeling dye (Green) (EXOGP300A‐1, System Biosciences, SBI) was added to the EV preparation. After incubation at 37°C with shaking (350 rpm) for 20 min, 167 μl of ExoQuick‐TC (EXOTC50A‐1, System Biosciences, SBI) was added to the solution and incubated overnight at 4°C, followed by centrifugation at 10,000 rpm for 10 min. Isolated ipRPE‐EVs stained with Exo Glow green were incubated with undifferentiated hiPSCs for 48 h. Finally, samples were fixed with 4% PFA and stained with Alexa Fluor 594 Phalloidin (A12381, ThermoFisher Scientific) for imaging using the Nikon C2 laser scanning confocal microscope.

### 2.20 Statistical analysis

Statistical analyses were performed using GraphPad Prism 7.0 software and shown as mean ± SD. Three biological replicates were used for analysis in all cases. The statistical significance of the difference was determined using Student *t*‐test, and the one‐way ANOVA with Tukey post‐test conducted for multiple comparisons. Significant differences were denoted with asterisks: *(*p*  < 0.05), **(*p*  < 0.01), ***(*p* < 0.001), ****(*p* < 0.0001). Bioinformatics and Proteomics expression analysis are described in their corresponding sections.

## 3 RESULTS

### 3.1 Derivation of human induced‐primary RPE from human retinal organoids

We previously established a protocol for efficient and reproducible generation of retinal organoids from human iPSCs (hRetOs) (Zhong et al., 2014). These hRetOs display spatial and temporal features that replicate the development of the human retina in vivo, including fully laminated neural retina and light‐sensitive photoreceptors. Importantly, as in the case of the native retina, retinal pigment epithelium (RPE) cells also develop in conjunction with the neural retina, giving rise to an RPE tissue continuous with the adjacent neural retinal epithelium and bundled at the tip of the hRetOs (Zhong et al., 2014). Building upon this system, we first sought to establish a simple and efficient strategy to derive human RPE monolayer cultures directly from hRetOs. The underlying rationale for the methodology we envisioned was as follows: provided the RPE tissue associated with the hRetOs consists of a polarised monolayer of RPE analogue to the native human RPE, RPE cultures could be derived from it by similar methods to those used for deriving cultures of primary human RPE cells. Thus, we initially undertook a thorough characterisation of the developmental and maturation features of RPE tissue associated to the hRetOs.

**FIGURE 1:**
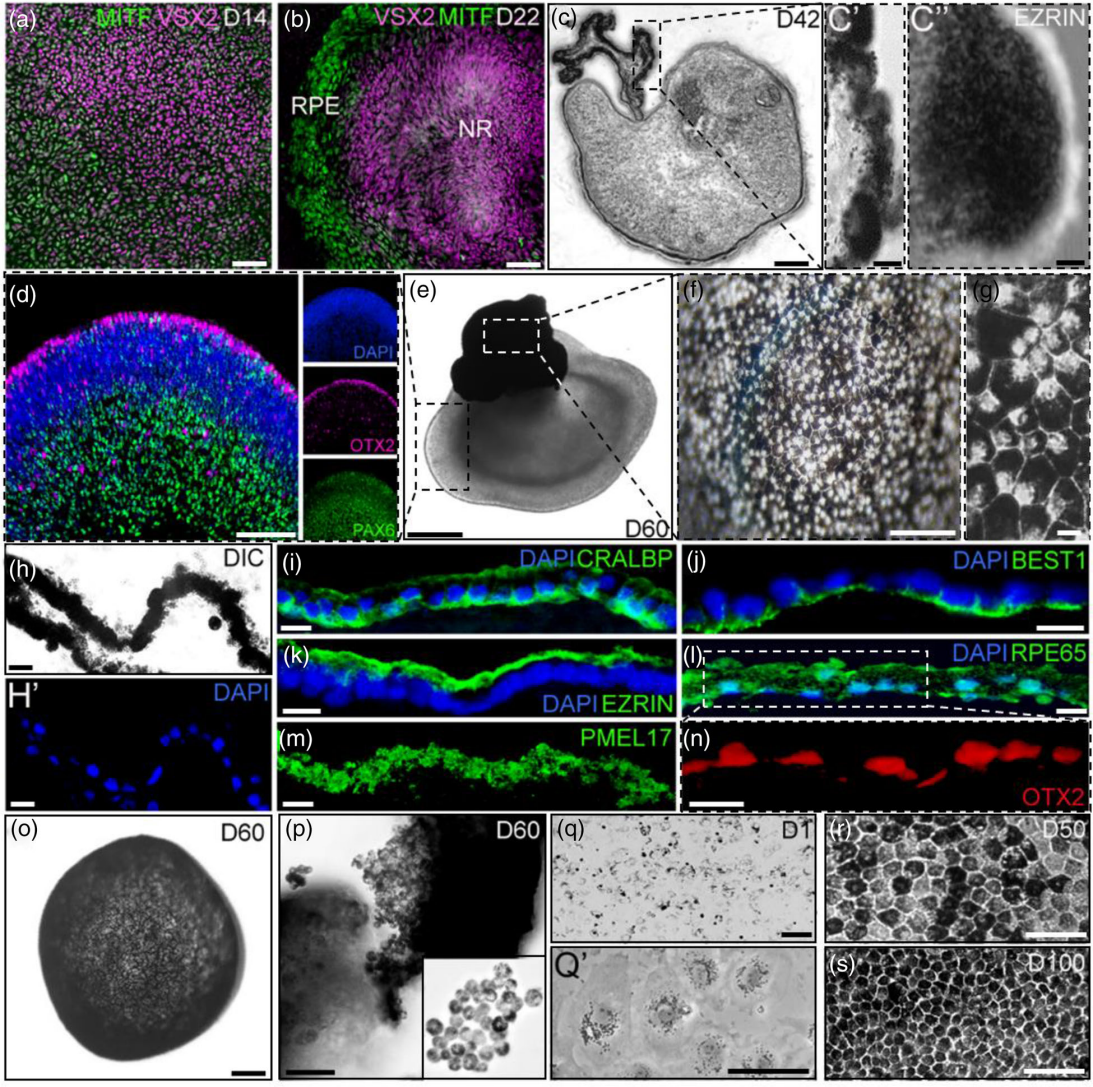
Derivation of induced‐primary RPE from human retinal organoids. At early stages of differentiation of hiPSC into retinal organoids, RPE progenitor cells showed a salt‐and‐pepper distribution within domains composed of bipotent MITF^(+)^/VSX2^(+)^ progenitors, MITF^(‐)^/VSX2^(+)^ NR progenitors and MITF^(+)^/VSX2^(‐)^ RPE progenitor cells (a). As differentiation progressed, cells differentiated into a central VSX2 positive NR domain and a peripheral RPE domain expressing MITF but not VSX2 (b). Detached NR domains cultured in suspension formed the characteristic hRetOs, composed of a transparent pseudostratified NR epithelium (c) continuous with the adjacent one cell thick layer of polarized pigmented RPE (c’‐c’’). Over time, hRetOs acquired the characteristic retinal lamination normally observed (d) while the RPE spheroid (e) appeared heavily pigmented (F) and exhibited the classic polygonal cell pattern (g). RPE contiguous to the hRetOs (h, h’) expressed key proteins associated to normal polarisation and function of the native RPE: CRALBP (i), BEST1 (j), EZRIN (k), RPE65 (l), PMEL17 (m) and Otx2 (n). Dissected RPE spheroids (o) dissociated into single cells (P) and seeded onto permeable Transwell (q, q’) gradually grew into a characteristic pigmented cobblestone monolayer (r, s). Scale bars, 2 μm (c’’); 10 μm (c’, G and I–N); 20 μm (h and h’); 50 μm (a‐b, o‐p and q’‐s); 100 μm (c‐f and q)

During normal eye development, cell‐fate specification into either neural retina (NR) or RPE is regulated critically by two transcription factors, VSX2 and MITF, which initially are coexpressed in the bipotent progenitor cells but subsequently become restricted to the NR and RPE, respectively (Adler & Canto‐Soler, 2007; Horsford et al., 2005; Nguyen & Arnheiter, 2000). As in the native situation, the cells in our cultures followed the same differentiation sequence. RPE precursor cells expressing MITF were widely observed on day 14 (D14) of differentiation, with a salt‐and‐pepper distribution within domains composed of bipotent MITF^(+)^/VSX2^(+)^ progenitors, MITF^(‐)^/VSX2^(+)^ NR progenitors and MITF^(+)^/VSX2^(‐)^ RPE progenitor cells (Figure 1a). As differentiation proceeded, and recapitulating the behaviour observed in vivo (Nguyen & Arnheiter, 2000), MITF^(+)^/VSX2^(‐)^ RPE precursor cells segregated from MITF^(‐)^/VSX2^(+)^ NR progenitor cells giving rise to an RPE domain peripherally to, and surrounding the, NR domain (Figure 1b), closely mimicking the topological organisation of these two domains as observed in the native state during human retina development (Cook et al., 1994; O'rahilly, 1975). Following individual mechanical isolation of the NR/RPE domains and culture in suspension as described in our previous study (Zhong et al. 2014) they formed the characteristic hRetOs with a transparent pseudostratified NR epithelium continuous with the adjacent RPE (Figure 1c–g; Figure S1A and B; and Zhong et al., 2014). Under these conditions, the RPE domain acquired a spheroid shape and became gradually pigmented, with evident pigmentation observed as early as day 25 of differentiation (D25) (Figure S1A and B) coincident with the time pigmentation in the RPE in the human embryo is first observed (Remington & Remington, 2012). Likewise, as also observed in the human native RPE (Remington & Remington, 2012), after 6 weeks of differentiation, the RPE in the hRetOs already formed a one cell thick layer composed of polarised pigmented cells with cuboidal to columnar shape (Figure 1c‐c’’). As differentiation continued, both the NR and the RPE components of the hRetOs presented a steady growth; by D60 of differentiation (8.5 weeks), the NR showed the characteristic layers normally observed in these hRetOs (Zhong et al., 2014) (Figure 1d) while the RPE spheroid appeared heavily pigmented (Figure 1e–g). Furthermore, the RPE maintained the typical monolayer organisation, and expressed key proteins associated with normal polarisation and function of the native RPE including RPE65, an isomerohydrolase critical for the regeneration of the visual pigment (Cai et al., 2009); BEST1, a calcium‐activated anion channel implicated in transepithelial fluid transport; EZRIN, a protein playing a determinant morphogenetic role in the maturation of RPE microvilli (Bonilha et al., 1999); PMEL17, a pigment cell‐specific protein responsible for the formation of fibrillar sheets within the pigment organelle, the melanosome (Watt et al., 2013); and CRALBP, a cellular retinaldehyde‐binding protein involved in the retinal visual cycle (Saari et al., 2001) (Figure 1h–n). Taken together these observations strongly support that the RPE tissue associated with the hRetOs is analogue to the native human RPE in its timing of differentiation, histological organisation, and key features of functional maturation.

We thus set to establish a strategy to derive human RPE monolayer cultures directly from the RPE tissue associated to the hRetOs by mimicking the approach for deriving cultures of primary human RPE cells (Blaauwgeers et al., 1999; Geisen et al., 2006; Hu & Bok, 2001; Maminishkis et al., 2006; Sonoda et al., 2009). Hence, the RPE spheroids were dissected out from the NR component of the organoids, enzymatically dissociated into single cells and seeded onto Transwell filters coated with Matrigel (Figure 1o–q’; Figure S1C–E). Under these conditions, the dissociated RPE cells emulated the behavior of primary human RPE cells gradually growing into a characteristic pigmented cobblestone monolayer (Figure 1q–s). Following this method, RPE cells can be reproducibly isolated and cultured as a monolayer from hRetOs at D35 onward. To increase the efficiency of the process by collecting a larger amount of RPE tissue from the hRetOs, we generally used D60 hRetOs (Figure 1f; Figure S1C and D). Similar results were obtained from three different hiPSC lines Gibco Human Episomal iPSC Line (female) (Burridge et al., 2011); a human primary neonatal fibroblast‐derived line (IN2–5, male) (Kogut et al., 2018); and a human primary dermal fibroblast derived from a 62‐year‐old female (ic4‐4) (Kogut et al., 2018), with consistent and reproducible derivation of RPE monolayer cultures expressing key functional genes (Figure S2). From this point forward, we will refer to RPE monolayers derived from hRetOs as induced‐primary RPE cells (ipRPE), with the time of RPE isolation as passage 0 (P0) (Figure 1o and p).

### 3.2 Dynamics of differentiation and functional maturation of human ipRPE monolayers

Twenty‐four hours after seeding, cells attached to the surface of the well showed an epithelioid morphology and appeared lightly pigmented with melanin granules aggregated around the nucleus (Figure 1q‐q’). After 2 days, cells reached confluency, their pigment density increased indicating new melanin synthesis, and their polygonal shape became more uniform, eventually achieving the pigmented hexagonal mosaic pattern characteristic of primary RPE cultures (Figure 1r and s; Figure S1E) (Maminishkis et al., 2006). Importantly, ipRPE cultures expressed genes and proteins that are key for proper RPE morphological and functional maturation throughout sequential subcultures, having similar expression profiles at the different passages (Figure S3A–C). Of note, on passage 4 (P4) we observed a continuous ipRPE monolayer covering the surface of the plate during the first 3 weeks of culture; by the end of the 4^th^ week however, the ipRPE was not a continuous monolayer, showing areas of confluent, mature RPE cells interrupted by acellular areas (Figure S3D). This is consistent with previous studies showing that stem cell‐derived RPE monolayers have altered phenotypes after four or more cell passages transitioning from an epithelial to a mesenchymal phenotype and eventually senescing (Croze et al., 2014; Singh et al., 2013). Thus, ipRPE cultures showed comparable dynamics of differentiation, long‐term maintenance of the pigmented cobblestone monolayer and expression of signature RPE genes and proteins (RPE65, BEST1, CRALBP, MITF, EZRIN, among others) during the first three passages. To define an optimal ipRPE passage for follow up studies, we carried out quantitative comparisons between P1 and P2 after 50 days of maturation. There were no significant differences in the level of expression of key RPE markers such as RPE65, BEST1, ZO‐1 and MITF (Figure S3E). ipRPE‐P1 reached higher transepithelial resistance (TER) levels compared to ipRPE‐P2 (P1: 473.76 ± 4.6 Ω/cm (Ambati et al., 2003); P2:363.62 ± 1.74 Ω/cm (Ambati et al., 2003; Figure S3F). In both passages however, the TER levels were consistent with measurements obtained in other studies using stem cell‐derived RPE, primary fetal human RPE, and primary adult human RPE cultures (Frambach et al., 1990; Maminishkis et al., 2006; Singh et al., 2013; Sonoda et al., 2009). In addition, P1 and P2 ipRPE cell passages were capable of polarised secretion of VEGF‐A (Figure S3G), which was predominantly secreted into the basal side as observed in primary fetal human RPE and primary adult human RPE cultures (Blenkinsop et al., 2015; Maminishkis et al., 2006). We noticed that we obtained five times higher yield of RPE cells with ipRPE‐P2 than ipRPE‐P1 (average 150,000–200,000 cells/well at P1 vs. 700,000–900,000 cells/well at P2; Figure S3H), while maintaining the RPE phenotype. These observations led us to select ipRPE‐P2 monolayers for all following experiments.

In an effort to establish a more in‐depth characterisation of the ipRPE‐P2 monolayers throughout differentiation, and to determine an optimal stage of maturation suitable for follow up studies, we carried out a time‐course analysis to evaluate expression of key RPE genes, cell morphology, pigmentation and ultrastructural and functional polarisation as reflected by transepithelial resistance (TER) and polarised secretion of bioactive molecules.

**FIGURE 2:**
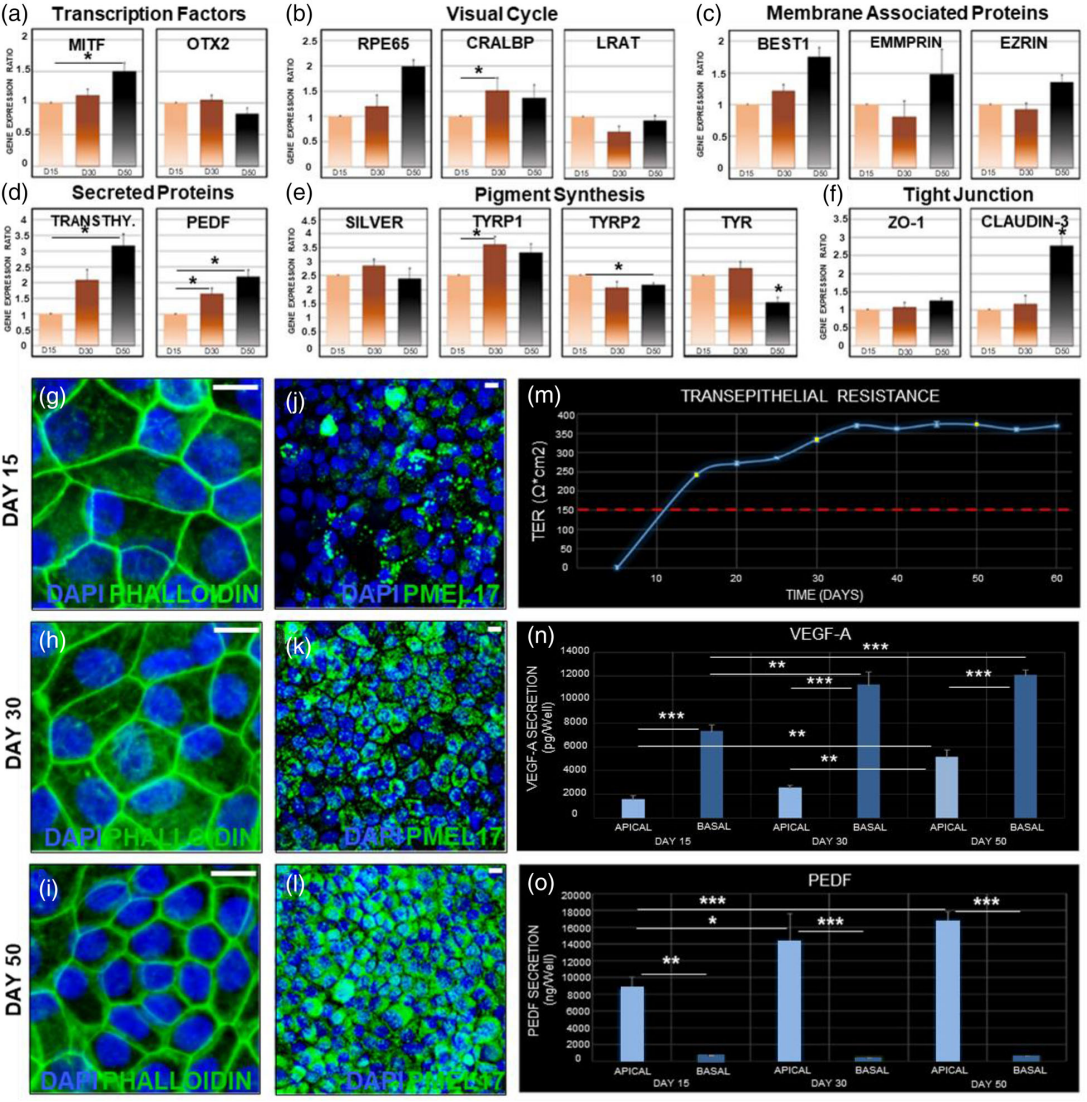
Time‐course analysis of differentiation and maturation of human induced‐primary RPE monolayers. Quantitative PCR at different developmental time points confirmed expression of genes involved in essential mechanisms of RPE, including gene regulation and fate specification (a), visual cycle process (b), ion channel function and cell polarisation (c), RPE secreted factors (d), pigment synthesis (e) and formation of tight junctions (f). Phalloidin staining showed the typical polygonal RPE cell shape, being more irregular in shape, larger in size and less tightly packed on D15 (g) compared to more mature ipRPE cells (D30, h) and finally displayed a regular firmly packed polygonal pattern characteristic of the native mature RPE (D50, i). PMEL17 (green) revealed a gradually increasing level of pigmentation through 15 (j), 30 (k) and 50 (l) days of differentiation. Functional polarisation as reflected by transepithelial resistance (m, yellow dots) and polarised secretion of bioactive molecules such as VEGF (N) and PEDF (o) also increased concomitantly with ipRPE differentiation. Bar graphs represent mean ± SD. *p < 0.05; **p < 0.005; ***p < 0.0005. Scale bars, 10 μm. *n* = 3

Quantitative PCR demonstrated expression of genes involved in essential mechanisms of RPE maturation and function, including gene regulation and fate specification (OTX2 and MITF), pigment synthesis (SILVER, Tyrp1, Tyrp2 and Tyr), formation of tight junctions (Claudin and ZO1), visual cycle process (RPE65, CRALBP and LRAT), ion channel function and cell polarisation (BEST1, EMMPRIN and EZRIN) and RPE secreted factors (PEDF and Transthyretin) in the ipRPE‐P2 cells (Figure 2a–f). Most of these genes showed a similar trend, being expressed early during the differentiation process, with detectable levels already at D15, and remaining relatively constant through D30, and D50 of differentiation. In some cases, the level of expression showed a slight or marked increase as the cells reached a more mature state (Figure 2a–f).

The shape and proper polarisation of individual RPE cells is maintained by their cytoskeleton components, in particular the actin microfilament network (Bonilha, 2014). Capitalising on the ability of phalloidin to selectively bind to F‐actin we confirmed normal morphological and polarisation differentiation in ipRPE‐P2 monolayers. Phalloidin staining revealed a classic polygonal RPE cell shape, with immature cells at earlier stages of differentiation appearing more irregular in shape, larger in size, and less tightly packed (Figure 2g) as compared with more mature ipRPE cells (Figure 2h and i). Over time, cells became smaller and with a more regular polygonal pattern resembling the honeycomb appearance of native mature RPE (Figure 2k). Also, consistently with previous studies, cell pigmentation showed a gradual progression (Buchholz et al., 2009; Klimanskaya et al., 2004; Maminishkis et al., 2006). Following subcultivation from P1 to P2, ipRPE cells initially showed very low pigmentation, with detectable granules of brown pigment in the cytoplasm of the ipRPE cells within 24 h after seeding, similar to that observed at P1 (Figure 1q and q’). The level of pigmentation gradually increased upon cell confluency, as shown by expression of the pigment protein PMEL17 on D15, D30 and D50 of differentiation (Figure 2j–l). By D50, PMEL17 appeared robustly expressed and densely packed all throughout the ipRPE monolayer (Figure 2l).

Polarisation of the RPE monolayer requires establishment of functional tight junctions between the cells, and leads to progressive increase in TER measurements as a consequence of the increase in electrical resistance across the RPE monolayer concomitant with cell maturation (Sonoda et al., 2009). Thus, we measured TER levels at the three time points chosen for our time‐course study (D15, D30 and D50) as a reliable indicator of ultrastructural polarisation and epithelial integrity. ipRPE‐P2 developed a TER of 250 Ω * cm^2^ within the first 2 weeks. Within 1 month in culture ipRPE‐P2 reached a TER of 320 Ω * cm^2^, soon thereafter reaching a plateau slightly above 350 Ω * cm^2^ that was consistently maintained over 60 days (Figure 2m). TER measurements obtained at D30 and D50 ipRPE monolayers were similar to the TER observed in previous studies in stem cell‐derived RPE and hfRPE, and significantly above the TER of 150 Ω * cm^2^ found in vivo (Singh et al., 2013; Sonoda et al., 2009; Vaajasaari et al., 2011; Wu et al., 2016).

We further evaluated functional polarisation of the ipRPE monolayer by determining the release of proteins known to be secreted preferentially from either the apical and basal side of RPE cells. To this end, we carried out quantitative comparisons between basal and apical release of vascular endothelial growth factor A (VEGF‐A) and pigment epithelium‐derived factor (PEDF), two factors known to act in choroidal neovascularisation and photoreceptor survival, respectively, favoring the photoreceptor/RPE/choroid symbiosis (Bhutto et al., 2006; Gao et al., 2001; Martin et al., 2004). Consistent with previous observations in primary hfRPE cultures and native RPE tissue (Maminishkis et al., 2006), VEGF‐A showed preferential secretion into the basal side (Figure 2n), while PEDF was mainly secreted towards the apical side (Figure 2o) (Patricia Becerra et al., 2004). Consistent with progressive ipRPE maturation, in both cases the level of protein secretion increased over time.

**FIGURE 3:**
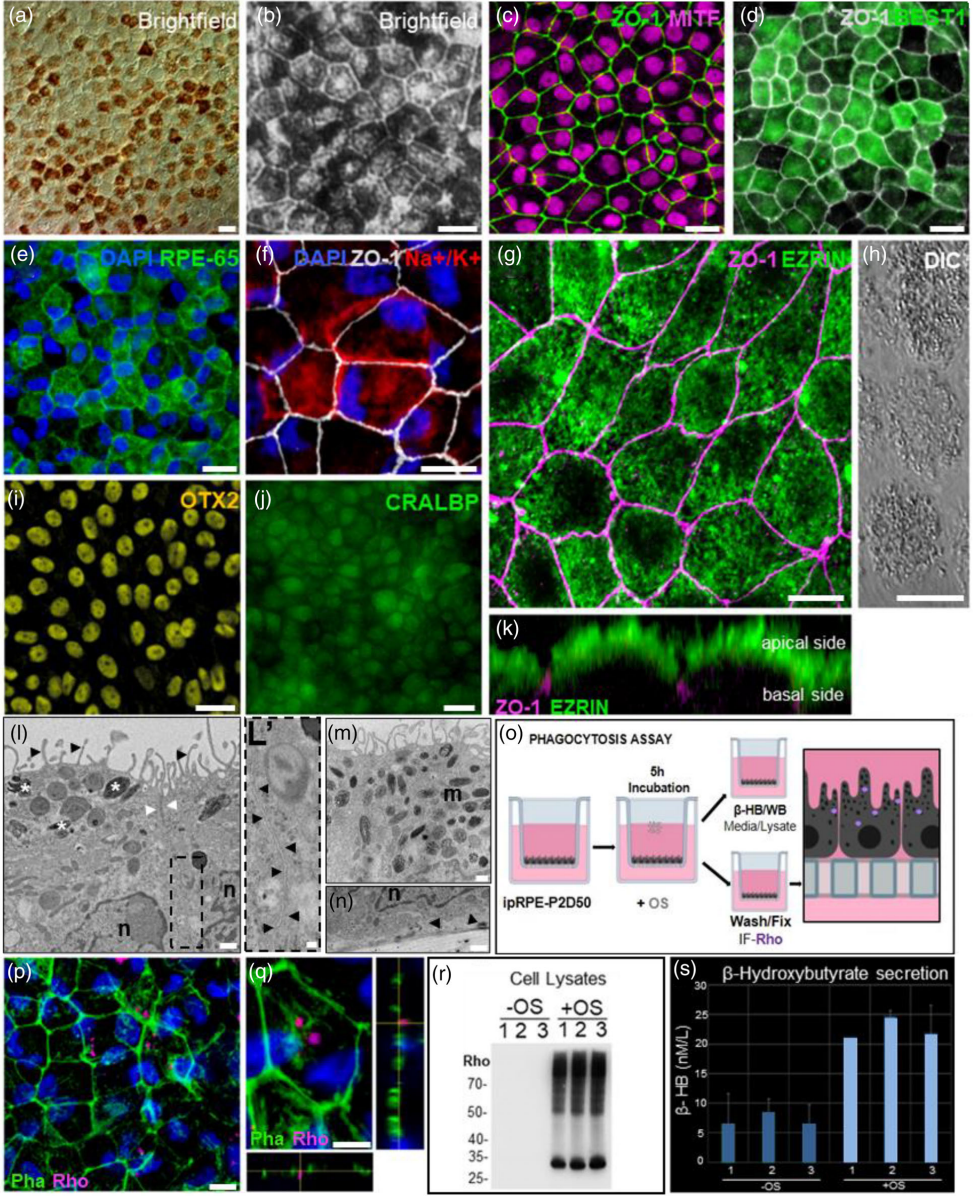
Induced‐primary RPE monolayers exhibited hallmarks of cell differentiation and key physiological characteristics of the native RPE tissue. Representative light microscopic images of passaged two (P2) ipRPE at day 50 (a & b). Cells expressed key RPE proteins ZO‐1 and MITF (c), BEST1 (d), RPE‐65 (e), Na+/K+ ATPase (f), EZRIN (g; punctate pattern reflecting its localisation within microvilli as shown by DIC in H), OTX2 (i) and CRALBP (j). Confocal orthogonal view revealed typical polarised expression of ZO‐1 (purple) and EZRIN (green) at the apical cell surface (k). Transmission electron microscopy showed abundant apical microvilli (l; black arrowheads), presence of tight junctions (l; white arrowheads) and adherent junctions (l’; black arrowhead), melanin granules at different maturation stages (m), basally localised nuclei, and characteristic basal infoldings corresponding to invaginations of the basal cell membrane (n; black arrowhead). To evaluate the capacity of the ipRPE‐P2 cells to phagocytose POS, cells were fed with POS isolated from bovine retinas (o). After 5 h ingestion of POS, the presence of rhodopsin within the RPE cells was confirmed by confocal microscopy (p‐q; immunolabelled rhodopsin in purple), and western blotting of RPE cell lysates (R). Increased levels of β‐HB further confirmed metabolisation of phagocytised POS by ipRPE cells (s). Scale bars, 20 μm (a–e and i–j); 10 μm (f–h); 5 μm (q); 500 nm (l‐n); 100 nm (l’). *n* = 3

After 50 days of differentiation, the ipRPE‐P2 monolayers showed evidence of advanced maturation at every level analyzed (Figure 3). At the tissue level, a continuous monolayer of tightly packed cells exhibiting a regular pigmented cobblestone pattern with a well‐established network of intercellular tight junctions as evidenced by labelling of Z01 protein (Figure 3a–c) was present. Furthermore, robust expression of key functional proteins including RPE65, BEST1, CRALBP, Na+/K+ ATPase and EZRIN – all showing their corresponding proper cellular localisation – was observed throughout the monolayer (Figure 3c–k). At the ultrastructural level, transmission electron microscopy showed abundant apical microvilli (Figure 3l, black arrowheads), the presence of tight junctions (Figure 3l; white arrowheads) and adherents junctions (Figure 3l’; black arrowhead) at the most apical side of the basolateral membrane, basally localised nuclei (Figure 3l and 3m), and characteristic basal infoldings corresponding to invaginations of the basal cell membrane (Figure 3n, black arrowhead). Classical football, needle‐like and oval‐shaped melanin granules at different maturation stages were abundant in the cytoplasm, mainly in the apical and mid‐parts of the cell but were absent from the basal cytoplasm (Figure 3l, white asterisks, and M, *m*).

At the functional level, as shown in Figure 2n and o, D50 ipRPE‐P2 monolayers displayed a native‐like balanced secretion of pro‐ and anti‐angiogenic factors, preferentially secreting VEGF to the basal side (choroid) and PEDF to the apical side (photoreceptors). In addition, D50 ipRPE‐P2 monolayers showed active phagocytosis of photoreceptor outer segments, a mechanism essential to support lifelong maintenance of photoreceptors in vivo (Strauss, 2005). ipRPE fed with photoreceptor outer segments (POS) isolated from bovine retinas (Figure 3o), internalised POS after 5 h, as evidenced by immunodetection of rhodopsin within the ipRPE cells (Figure 3p and q). The uptake of rhodopsin was also confirmed by western blotting of ipRPE cell lysates exposed to POS (Figure 3r). Furthermore, the main lipids in POS are phospholipids, which upon phagocytosis are digested liberating fatty acids, which are then utilised by the mitochondria to generate beta‐hydroxybutyrate (β‐HB) (Reyes‐Reveles et al., 2017). Consistently, levels of β‐HB were over 2‐fold in ipRPE cells incubated with POS (Figure 3s).

Confocal three‐dimensional volume rendering of D50 ipRPE‐P2 monolayers further confirmed the advanced degree of polarisation and functional maturation achieved by the cells, as evidenced by the core bundle of densely packed actin filaments within the microvilli, the circumferential actin belt associated to the adherent junctions, and actin filaments associated to the basal infoldings at the most basal side of the cells (Movie S1), as well as the apical localisation of the melanin granules (Movie S2), as observed in fully mature RPE cells (Bonilha, 2014). Likewise, internalisation of phagocytosed POS was further documented (Movie S3).

Collectively, these results demonstrate that our ipRPE‐P2 cultures exhibit hallmarks of cell differentiation and key physiological characteristics of the native tissue and its derived primary cultures. Furthermore, after 50 days in culture ipRPE monolayers are fully polarised, and exhibit functional hallmarks of bona fide mature RPE cells, providing a suitable system to mimic the biology and function of the native human RPE. Therefore, all experiments performed from this point forward were done using D50 ipRPE‐P2 monolayers.

### 3.3 ipRPE under homeostatic conditions constitutively releases EVs

**FIGURE 4:**
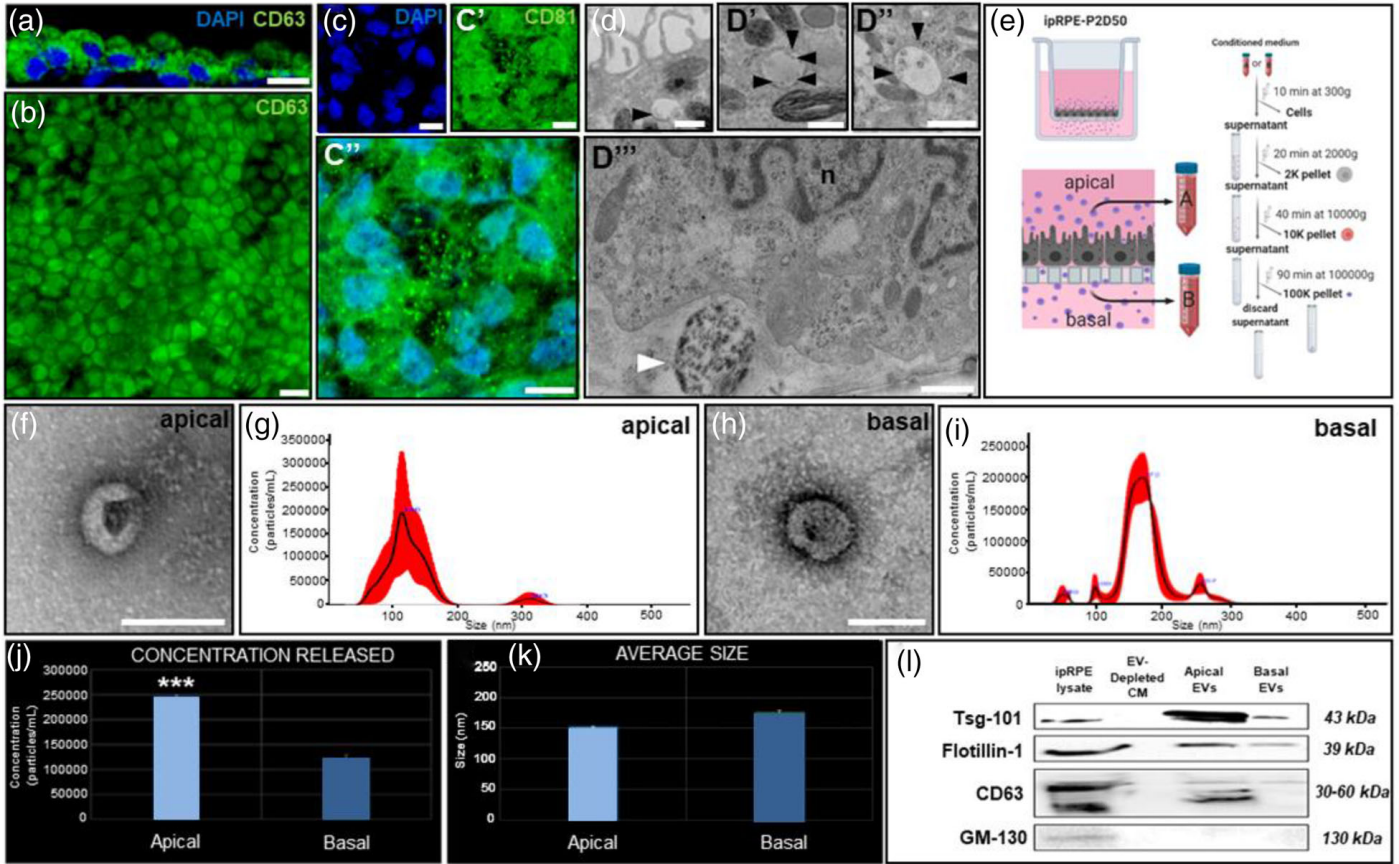
Isolation and characterisation of induced‐primary RPE‐derived extracellular vesicles (EV). Classical EV biomarker CD63 was identified in the cytoplasm of both, the RPE growing attached to the hRetOs (a) and the ipRPE monolayers (b). CD81, another EV indicator, showed cytoplasmic staining and prominent expression in punctate structures at the apical surface (c). Ultrastructural analysis of ipRPE monolayers revealed the presence of endosomes and multivesicular bodies (black arrowheads) intermingled with melanosomes in the apical side (d‐d’’), and near the basal infoldings of the cells (d’’’, white arrowhead). EVs were isolated from either apical‐*A* or basal‐*B* conditioned media (CM) by differential ultracentrifugation as shown in the diagram (e). Typical clear rounded membrane vesicles within a size range of 50–200 nm were observed by TEM of EV pellets from both apical (f) and basal side (h). Nanoparticle tracking analysis revealed the size distribution for the apical (g) and basal (i) sides, the average concentration and, average size of EVs released by ipRPE monolayers (k). Western blots against well‐known EV specific markers (TSG‐101, Flotillin‐1 and CD63) and an intracellular Golgi membrane protein (GM130) confirmed the purity of EV preparations obtained from ipRPE monolayers (l). Bar graphs represent mean ± SD. ***p < 0.0001. Scale bars, 10 μm (a and c); 20 μm (b); 500 nm (d–d’’’); 100 nm (f and h). *n* = 3

EVs contain specific sets of lipids, proteins, DNA and RNAs (Théry et al. 2018) and have been rendered important physiological functions as well as potential for diagnostic and therapeutic applications (Hoshino et al., 2020, Lee et al., 2012). RPE cells are known to release EVs (Klingeborn et al. 2017) but their possible role in retinal physiology and disease is still mostly unknown (Klingeborn et al., 2018). To better understand the role of EVs within the dialogue between photoreceptors, RPE and choroid, we decided to isolate and characterise EVs released from our ipRPE system. Members of the tetraspanins (TSPAN) family CD63 and CD81 are the most frequently identified proteins in EVs, being considered classical EV biomarkers (Andreu & María Yáñez, 2014). Intracellularly, both are predominantly localised to late endosomes and lysosomes (Hosokawa et al., 2020; Van Niel et al., 2011), while CD81 is also expressed at the apical surface of the RPE (Chang & Finnemann, 2007). Consistently, a high expression of CD63 was found in the cytoplasm of both, the RPE growing attached to the hRetOs and the ipRPE monolayers (Figures 4a and 4b) while CD81 showed cytoplasmic staining and prominent expression in punctate structures at the apical surface (Figure 4c and Movie S5). Moreover, ultrastructural analysis of ipRPE monolayers revealed the presence of fused vesicles and vacuoles. Potential endosome and multivesicular bodies, containing EVs, are located near the basal infoldings of the ipRPE cells and between the melanosomes in the apical area (Figures 4d‐d’’’, black and white arrowheads). Furthermore, EVs secreted from ipRPE monolayers from either apical or basal conditioned media (CM) were successfully isolated by differential ultracentrifugation (Figure 4e), the generally preferred technique for EV separation and concentration (Théry et al., 2018). Characteristic clear rounded membrane vesicles within a size range of 50–200 nm were observed by transmission electron microscopy (Figures 4 f‐apical & h‐basal). Nanoparticle Tracking Analysis (NTA) demonstrated EV size distribution with a peak at 146 nm for the apical side and 172 nm for the basal side (Figures 4g & i). The average concentration of EVs corresponded to 2.43 × 10^5^ ± 5.70 × 10^3^ particles/ml at the apical side and 1.17 × 10^5^ ± 1.20 × 10^4^ particles/ml at the basal side (Figure 4j). EV mean diameters for apical and basal side were 153 ± 5 nm and 173.5 ± 5.4 nm respectively (Figure 4k). Immunoblotting against well‐known EV specific markers (TSG‐101, Flotillin‐1 and CD63) as well as GM130, a cis‐Golgi membrane protein not expected to be enriched in EVs, confirmed the purity of our EV preparations. EV markers were present in EV samples and ipRPE cell lysates but absent from EV‐depleted CM (Figure 4l). Moreover, GM130 was not detected in EV preparations and EV‐depleted CM although it was present in the corresponding ipRPE cell lysates, indicating that the EV preparations were not contaminated by cellular debris (Figure 4l). Thus, in accordance with MISEV2018 guidelines (Théry et al., 2018), the physical characteristics of the ipRPE‐derived vesicle preparations (ultrastructural morphology and size), and their biochemical composition (presence of Flotillin‐1, Tsg101, CD63 and absence of GM130), confirmed that they fulfill the criteria for EVs including exosomes and microvesicles (Théry et al., 2018). From this point forward, we will refer to EVs isolated from our ipRPE as ipRPE‐EVs.

Time‐course analysis through ipRPE differentiation (15, 30 and 50 days) revealed that the concentration of EVs released from ipRPE monolayers decreased concomitantly with RPE differentiation and maturation (Figure S4I), consistent with previous observations of EVs dynamics during aeging (Noren Hooten, 2020). In addition, NTA analysis revealed that the mode of the diameter of EVs recovered from the apical side was 179.2 ± 19.4 and 175.5 ± 6.4 nm for the basal side (Figure S4J). A sample of Brownian motion exhibited by ipRPE‐EVs in solution is provided in Movie S6.

**FIGURE 5:**
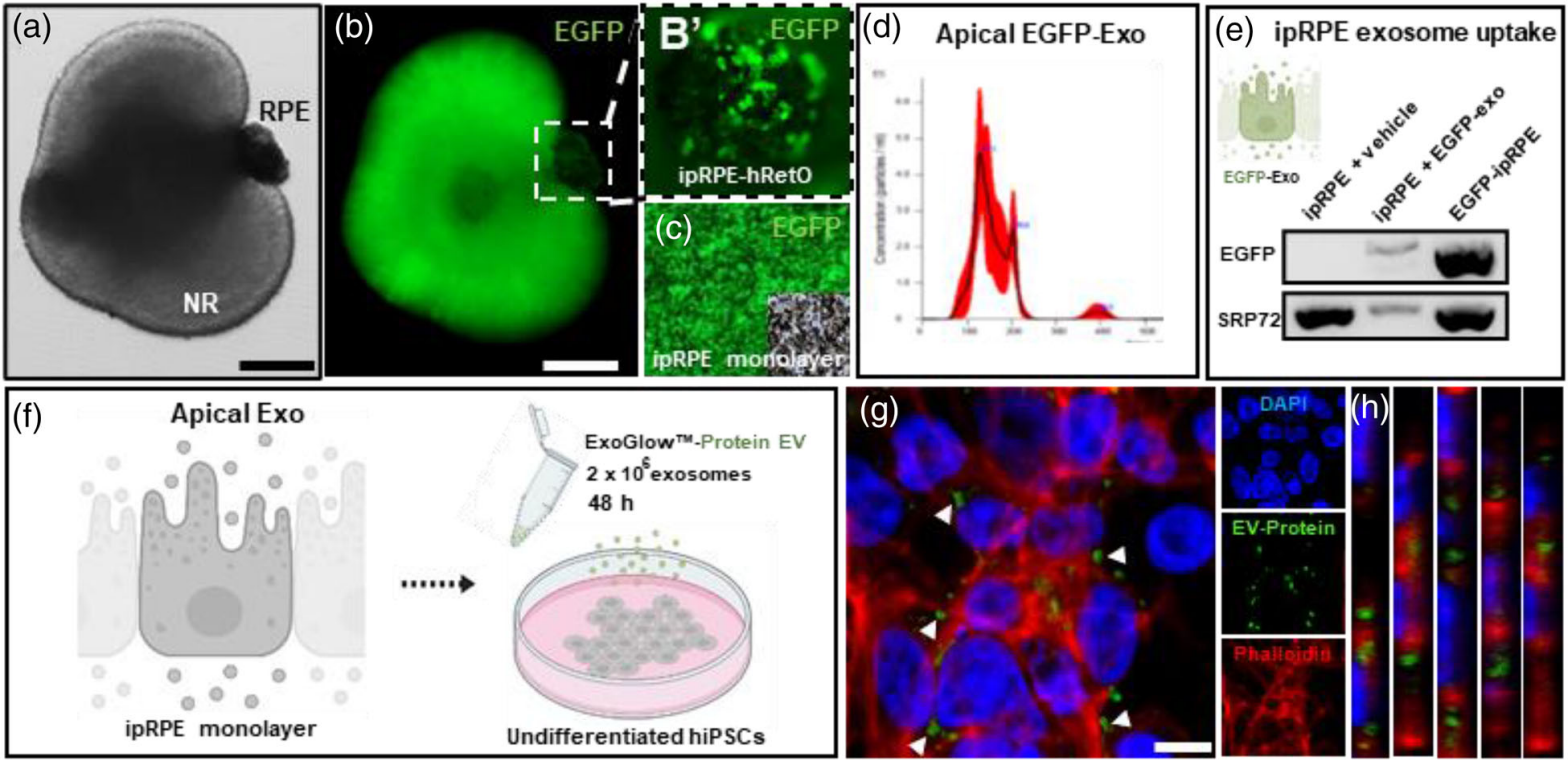
EV proteins and mRNA released from ipRPE cells are taken up by target cells. Transgenic EGFP hRetOs as shown by light (a) and fluorescence microscopy (b) with the adjacent pigmented RPE (b’) were used to derive EGFP‐ipRPE monolayers (c). Nanoparticle tracking analysis revealed the size distribution for apically secreted EVs by EGFP‐ipRPE monolayers (d). Transferred EGFP mRNA to non‐EGFP‐ipRPE cells was determine by RT‐PCR (SRP72 internal control gene and EGFP‐ipRPE cells positive control; e). Isolated ipRPE EVs stained with ExoGlow‐green EV protein dye were incubated with undifferentiated hiPSCs for 48 h (F). Phalloidin (red) was used to stain hiPSCs cytoplasm (g and h). Confocal three‐dimensional image of hiPSCs (g, including small panels for DAPI in blue, EV‐Protein in green and Phalloidin in red) and orthogonal views (h and h’) confirmed the internalisation of labelled EVs within the cytoplasm. Scale bars, 100 μm (a & b); 10 μm (g)

Next, to assess functional ability of ipRPE‐EVs to transfer cargo into target cells, we generated ipRPE monolayers expressing cytoplasmic EGFP (Figure 5a–c) and isolated apically secreted EGFP‐ipRPE‐EVs (Figure 5d). ipRPE‐EV uptake was confirmed by EGFP mRNA transfer from EGFP‐ipRPE‐EVs into WT (no‐EGFP) ipRPE cells after 48 h in coculture (Figure 5e). Furthermore, we also confirmed functional transfer of ipRPE‐EV protein cargo into target cells. For this, isolated ipRPE‐EVs stained with ExoGlow‐green EV Protein Dye were incubated with undifferentiated hiPSCs for 48 h (Figure 5f). The fluorescent green dye generated a robust signal specific for internal EV proteins allowing clear identification of EVs internalised in the recipient cells (Figure 5g). Confocal images and orthogonal views further confirmed transfer of protein cargo from ipRPE‐EVs into the cytoplasm of recipient cells (Figure 5h).

### 3.4 ipRPE‐EVs contain proteins involved in AMD pathogenesis and show apical‐basal directional proteome enrichment

**FIGURE 6:**
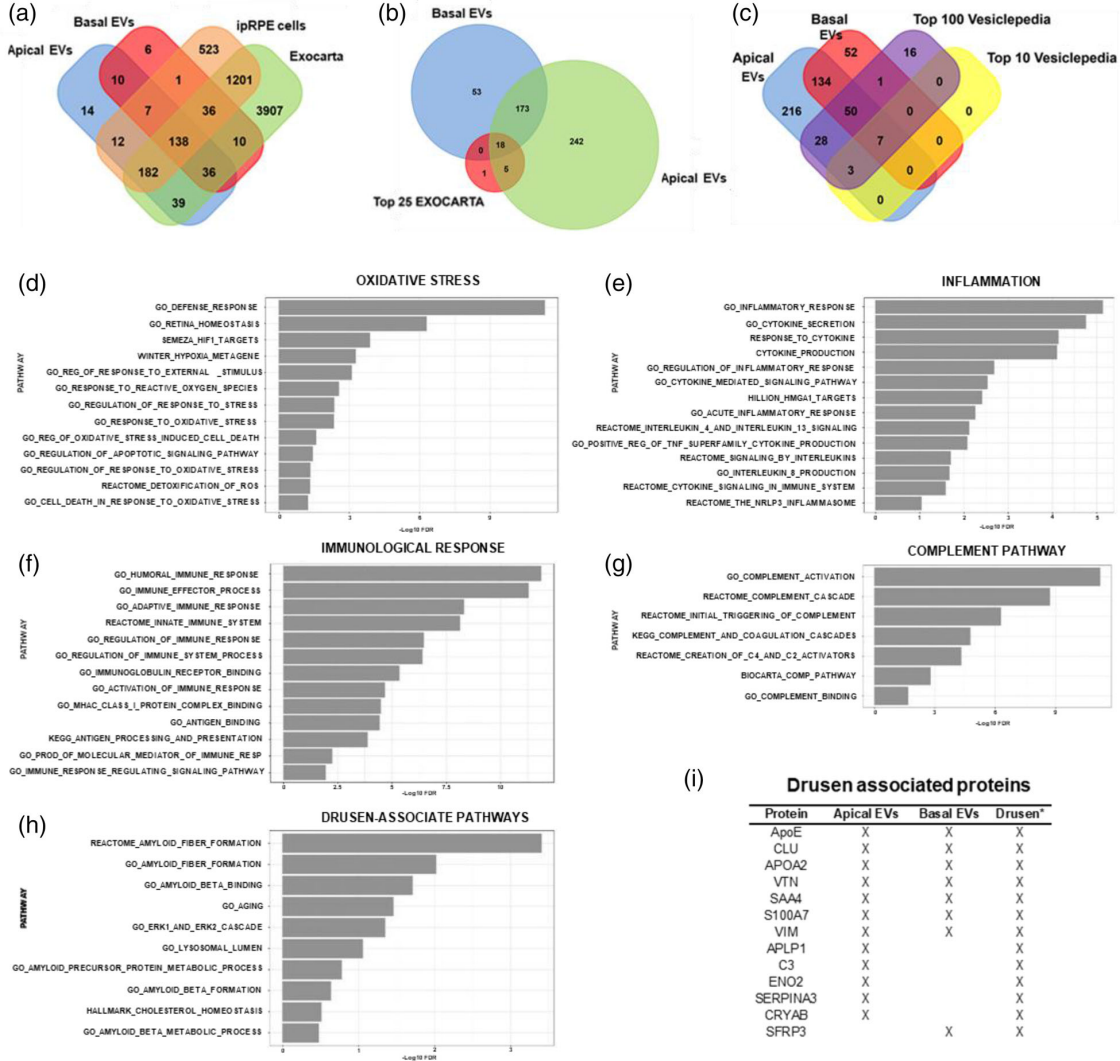
Proteomics analysis of ipRPE monolayers and their released extracellular vesicles. (a) Purified extracellular vesicles from ipRPE monolayers were subjected to mass spectroscopy and protein levels compared to parent ipRPE cellular levels. Venn diagram showing total detectable spectral counts found in ipRPE cells, EVs (Apical or Basal), or both; 3% of human proteins identified in ipRPE‐EVs have not been previously documented to exist in EVs . (b‐c) Venn diagrams displaying the proteins identified in apical and basolateral EVs from ipRPE monolayers contrasted to the Top 25 EXOCARTA (b), Top 10 Vesiclepedia and Top 100 Vesiclepedia (c) databases. (d‐i) Enrichment analysis of proteins identified in apical and basal EVs revealed a significant enrichment in pathways associated to oxidative stress (d), inflammation (e), immunological response (f), complement (g) and drusen (h). Key proteins previously identified in human drusen were found in both apical and basal EVs released from ipRPE cells (I, * refers to Crabb et al. (2002)). ApoE: apolipoprotein E; CLU: clusterin; APOA2: apolipoprotein A‐II; VTN: vitronectin; SAA4: serum amyloid A‐4; VIM: vimentin; APLP1: amyloid‐like protein 1; C3: complement 3; ENO2: gamma‐enolase; SERPINA3: alpha‐1‐antichymotrypsin; CRYAB: alpha‐crystallin B; and SFRP3: secreted frizzled‐related protein 3. *n* = 3

To further characterise the composition of ipRPE‐EVs we examined their proteome cargo by performing liquid chromatography–mass spectrometry analysis on ipRPE cells and ipRPE‐EVs secreted from both apical and basal side. Proteomic analysis identified a total of 2215 proteins; of these, 1724 were found only in ipRPE cells, 376 in both ipRPE cells and ipRPE‐EVs and 115 detected only in ipRPE‐EVs (Figure 6a). A total of 481 different proteins were identified in apical and basolateral EVs, of which 247 were found exclusively in apically released EVs and 53 exclusively in basolateral secreted EVs (Figure 6a). This differential distribution supports a directional enrichment mechanism of protein sorting and secretion in RPE tissue via EVs.

First, we cross‐referenced the ipRPE‐EV data set with two publicly available EV proteome databases: Exocarta, a primary resource of exosomal proteins, RNAs and lipids (http://www.exocarta.org) (Mathivanan et al., 2012); and Vesiclepedia, a compendium of RNA, proteins, lipids and metabolites in EVs (http://microvesicles.org) (Pathan et al., 2019). Our analysis indicated that 90% and 97% of identified human proteins, apically and basolaterally released, respectively, have been previously described in exosomes. We also identified 67 additional proteins (43 apical and 24 basal) that were not previously documented to exist in exosomes (Figure 6a). Likewise, we contrasted the ipRPE‐EV protein cargo to the top 25 proteins most often identified in exosomes (Mathivanan et al. 2012) (Figure 6b), of which 24 matched. Furthermore, ipRPE‐EVs contained all Top 10 Vesiclepedia proteins and 95 of the Top 100 Vesiclepedia proteins (Figure 6c).

Further bioinformatic analysis was performed to correlate ipRPE‐EV proteins with predicted biological processes. Within the set of proteins identified in apical and basal ipRPE‐EVs we determined the percent distribution of associated molecular processes. The analysis showed that ipRPE‐EV proteins were mostly related to the processes of cell communication, signal transduction, cell growth and/or maintenance, protein metabolism, energy pathways, metabolism, transport, regulation of nucleobase, nucleoside, nucleotide and nucleic acid metabolism, immune response and cell adhesion (Figure S5A–D).

Finally, to gain functional insight on ipRPE‐EV proteome cargo, we conducted over representation analysis (Yu et al. 2012) to determine whether known biological functions or processes are enriched in an experimentally‐derived gene list. We used the Gene Ontology (GO), Kyoto Encyclopedia of Genes and Genomes (KEGG) and Hallmark collections (Liberzon et al. 2011) in proteins released from apical and basal EVs. To understand the functional significance of identified proteins after protein enrichment, pathways were manually classified according to their link to various biological processes, molecular functions and cellular components (Table S1). Our analysis revealed a significant enrichment in pathways such as oxidative stress, inflammation, immunological response, complement pathway and drusen associated pathways (Figure 6d–h) which are highly involved and influenced during retinal disorders (Anderson et al., 2002; Beatty et al., 2000; Faber & Nissen, 2008; Jha et al., 2006; Nita & Grzybowski, 2016; Whitcup et al., 2013). Other pathways of interest (*p*‐value < 0.05) are sorted and summarised in Table S1.

Excitingly, some of the key proteins known to contribute to drusen formation during AMD (Crabb et al., 2002; Wang et al., 2010) such as apolipoprotein E (ApoE), apolipoprotein A‐2 (APOA2), clusterin (CLU), vitronectin (VTN), vinculin (VIN), complement 3 (C3) and alpha‐crystallin B (CRYAB) were also found in our ipRPE‐EVs (Figure 6i).

In addition, identified proteins in ipRPE‐EVs also included members of several families related to both, RPE normal and pathologic physiology: complement cascades (C4a, C4b, CFI, CFB, C1r, C1QBP, C1S, C3 and C1qTNF); histones family (H2A2B, H2B1D, H2B1O, H2A2C, H2A1B and H2AZ); immunoglobulins (IGHG1, IGHG2, IGHG3, IGHG4, IGKC, KV203, KV302, KV402, IGLC2, LV302 and LV403); annexin family (ANXA1, ANXA2, ANXA3, ANXA4, ANXA5, ANXA6, ANXA7 and ANXA11); heat shock protein family (HspA8, HspD1, HspE1, Hsp71, Hsp72, Hsp90A, Hsp90AA4P, Hsp90B, Hsp90B1, GRP75, GRP78); cytoskeletal proteins (cytokeratin 1, 2, 4, 5, 6 A, 6C, 7, 8, 10, 13, 16, 17, 18, 78 and 80); key RPE proteins (PEDF, EZR, TTHY, TYRP1, TYRP2, PMEL, ATP1A3, EMMPRIN and VEGF); myosin family (MYH3, MYH8, MYH9, MYH10, MYH14 and MYL6); ankyrin family (POTEF and POTEJ); cathepsin family (CTSL2, CTSC, CTSD, CTSH and PCDGI); antioxidant family (SOD1, TXN, PRDX1, PRDX4, GSTP1); S100 proteins (S100A6, S100A7, S100A8, S100A9, S100A10, S100A11 and S100A13); extracellular matrix proteins (Collagen alpha, Laminin alpha, beta and gamma); and other groups listed in Table S2.

Therefore, our proteomic analysis revealed that ipRPE‐EV protein cargo is significantly enriched in pathways associated to AMD pathogenesis, including oxidative stress, immunological response, inflammation, complement activation and notably drusen composition (Ambati & Fowler, 2012; Bok, 2005; Boya et al., 2016; Datta et al., 2017; Mitter et al., 2014).

### 3.5 Chronic oxidative stress leads to increase of drusen associated proteins in ipRPE‐EV cargo

**FIGURE 7:**
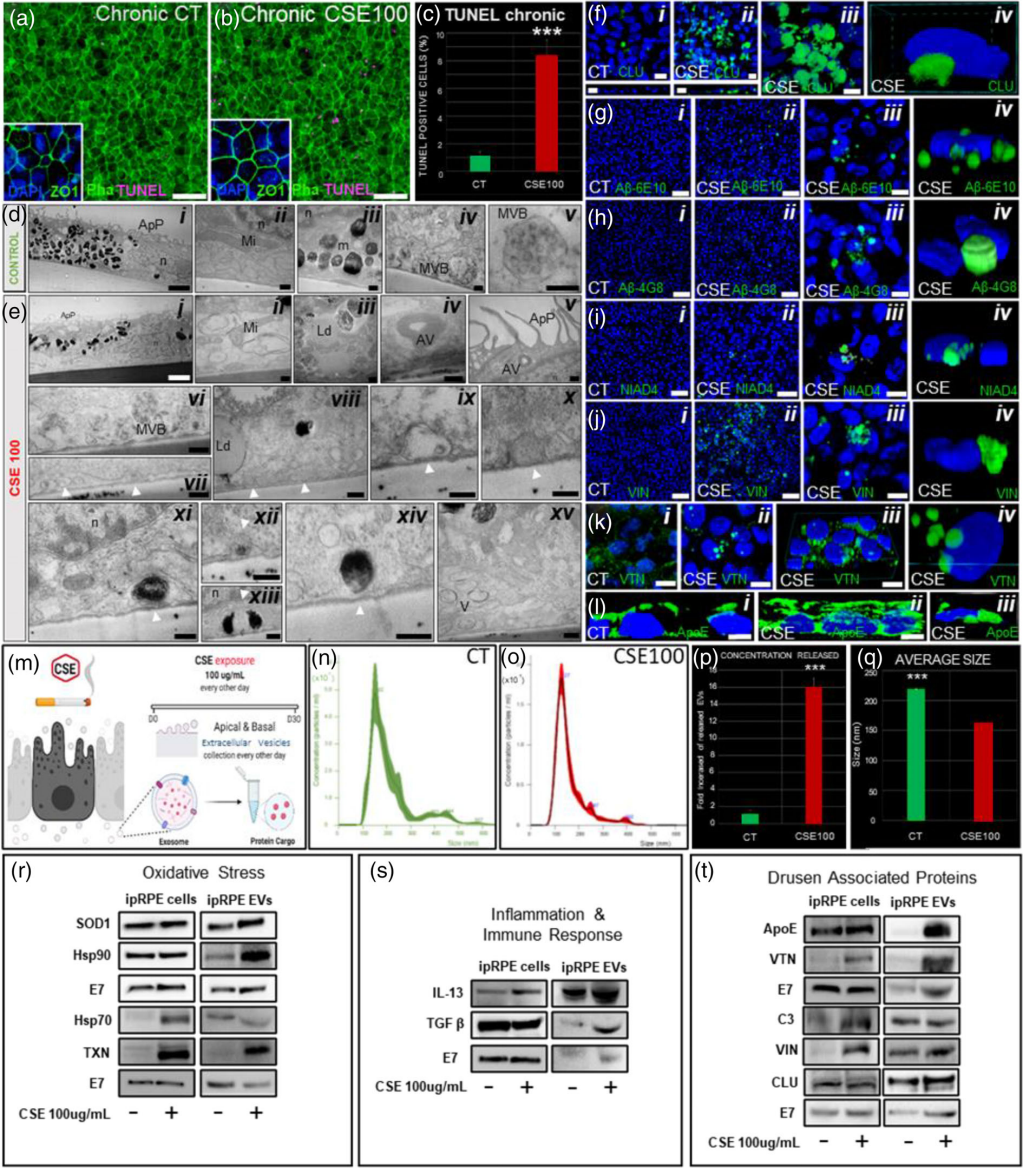
Chronic exposure to cigarette smoke in ipRPE cells. TUNEL on non‐treated ipRPE monolayers (a) and, after chronic CSE exposure (b, 100 ug/ml) was performed to identify apoptotic cells (purple in a‐b). Quantitative analysis revealed significant increase of apoptotic cell death after chronic CSE (c). Barrier properties of ipRPE monolayers were also evaluated by staining with ZO‐1 (a & b insets). Ultrastructural morphology of non‐treated ipRPE monolayers (D) showing apical microvilli (d, i), mitochondria (d, ii), melanosomes (d, iii) and the presence of endosome and multivesicular bodies containing extracellular vesicles (d, iv & v). Ultrastructural morphology of ipRPE cells under CSE oxidative insult (e) showing similar phenotypic manifestations as those found in AMD, including reduced cytoplasm (e, i), dilated mitochondria (e, ii), lipid droplets (e, iii), autophagosomes (e, iv and v), endosome and multivesicular bodies (e, vi), sub‐RPE basal deposits or drusen‐like deposits (e, vii‐xiv, white arrows), and, numerous vacuoles, which were eventually associated with clusters (e‐xv). Immunofluorescence analyses of ipRPE sections and flat mounted ipRPE cell cultures confirmed accumulation of CLU (f), Aβ (6E10 (g), 4G8 (h) and NIAD‐4 (i)), VIN (j), VTN (k) and ApoE (l) on the basal side of CSE‐treated ipRPE monolayers compared to control (CT). Diagram of experimental design to assess effect of chronic CSE exposure on ipRPE‐EVs concentration release and protein cargo (m). Nanoparticle tracking analysis revealed the size distribution for the control (n) and CSE‐treated ipRPE cells (o), the average concentration of EVs (p) and, the average size of released EVs (q). Western Blot analysis of EV cargo identified proteins involved in key processes associated to AMD: oxidative stress (r), immunological response and inflammation (S), and drusen‐associated proteins (t). Bar graphs represent mean ± SD. ***p < 0.0005. Scale bars, 50 μm (a‐b, g i‐ii, h i‐ii and j i‐ii); 10 μm (f i‐iv, g iiii, H iii, i iii, j iii and k i‐iii); 5 μm (l i‐ii); 2 μm (d i and e i); 200 nm (d ii‐iv and e ii‐xv). Mitochondria (Mi), autophagosome structure (AV), multivesicular bodies (MVB), apical processes (ApP), melanosomes (m), lipid droplets (Ld), Vacuoles (V) and Nuclei (n). *n* = 3

As noted above, our proteomic analysis revealed that ipRPE‐EV cargo is associated to cellular pathways involved in AMD pathophysiology, including drusen composition. Interestingly, the EV marker CD63 has been found in drusen in AMD (Wang et al., 2009), further stressing the need to investigate a potential role of RPE‐derived EVs during AMD. To aid in this goal, we set to establish an ipRPE model of chronic oxidative stress capable of recreating hallmarks of AMD pathogenesis. Cigarette smoke is the single most important environmental risk factor for AMD (Thornton et al., 2005; Tomany et al., 2004), and a commonly used agent to induce RPE oxidative stress (OS). Thus, we first evaluated the effect of acute exposure (24 h) to CSE at different concentrations (0, 50, 100 and 200 ug/ml) to find a proper dose to induce oxidative stress without leading to widespread RPE cell death. CSE doses of 100 and 200 ug/ml led to 7% and 10% cell death, respectively (Figure S6) concomitantly with a concentration‐dependent increase in ROS (Figure S6). These results were consistent with previous studies in ARPE‐19 cells and hiPSC‐derived RPE, where 100 ug/ml CSE led to ROS production without compromising functional tight junctions and proper RPE polarisation (Dalvi et al., 2019; Wang et al., 2014). Thus, this concentration was selected for chronic CSE exposure conditions. Our observations confirmed that chronic CSE exposure did not cause widespread toxicity, with an 8% cell death rate, comparable to that observed in the acute exposure (Figure 7a–c), and without compromising the barrier properties of ipRPE monolayers as indicated by normal tight junction protein expression (ZO‐1; Figure 7a and b insets).

On the other hand, chronic CSE exposure led to the appearance of hallmarks of the early AMD phenotype (Bianchi et al., 2013; Galloway et al., 2017; Golestaneh et al., 2017; Sarks et al., 1988; Young, 1987). TEM assessment of ipRPE ultrastructural morphology revealed similar phenotypic manifestations as those found in AMD. Presence of sub‐RPE basal deposits or drusen‐like deposits was observed, which appeared as circular electron‐dense structures present extracellularly and underneath the ipRPE cell membrane (Figure 7 evi–xv); at higher‐magnification these deposits further demonstrated membrane‐displacing basal deposits (Figure 7 evii–xiv). Furthermore, ipRPE monolayers subjected to chronic CSE exposure showed dilated mitochondria, with a spherical‐shape and clear matrices with fewer crests (Figure 7eii), in contrast to non‐treated cells showing long mitochondria with extensive ramifications and dense matrices with tubular crests (Figure 7dii). Another observed feature was the frequent presence of vacuoles with heterogeneous content, varying greatly in electro‐density and including multilamellar aspects which usually are referred as autophagosomes (Figure 7eiv). Even though CSE‐treated cells showed reduced cytoplasm, no differences in core size, number of nucleoli or nuclear invaginations could be established (Figure 7di and ei). RPE cells under CSE presented an increase in the number of cytoplasmic vacuoles basolaterally located (Figure 7evii–x and xv). Another recurring element were lipid droplet accumulations or electron‐lucent droplets in CSE‐treated cells (Figure 7e iii and viii). Finally, reduced melanosomes were found in CSE‐treated cells when compared to non‐treated cells (Figure 7di and Ei). Consistent with the observed drusen‐like deposits by TEM, immunofluorescence detection of drusen associated proteins in sections and flat‐mounted samples of CSE‐treated ipRPE cultures revealed significant aggregate accumulation of CLU, Aβ (6E10, 4G8 and NIAD‐4), VIN, VTN and ApoE on the basal side of CSE‐treated ipRPE monolayers (Figure 7f–l). In particular, immunoreactivity for Aβ and ApoE revealed remarkable accumulation of Aβ deposits (Figure 7g–i) and significant increased expression of ApoE across the cytoplasm of ipRPE cells, including basolateral aggregates below the nuclei (Figure 7l).

Having established a model that combines several key aspects of AMD: chronic oxidative stress induced by cigarette smoke exposure, advanced age (ipRPE between D60‐D90) (Galloway et al., 2017), and drusen‐like deposits, we used this experimental platform to evaluate the effect of oxidative stress induced‐AMD phenotype in ipRPE‐EV secretion and protein cargo (Figure 7m). Chronic oxidative stress induced a 15‐fold increase in overall secretion of EVs (Figure 7p) with EV size distribution peaks at 152 nm (217.3 ± 2.2 nm mean diameter) in controls compared to 127 nm (160.5 ± 1.6 nm mean diametre) for chronic oxidative stressed ipRPE (Figures 7n, o and q). We then carried out semiquantitative Western Blot analysis for proteins involved in pathways associated to AMD pathophysiology and that were enriched in ipRPE‐EVs, including oxidative stress, immunological response, inflammation and drusen‐associated proteins. Antioxidant enzymes (superoxide dismutase 1 (SOD1) and thioredoxin (TXN)) and members of the heat shock proteins (Hsps) family (Hsp70 and Hsp90) showed increased expression in ipRPE cells (TXN and Hsp70) and ipRPE‐EVs (SOD1, TXN, Hsp70 and Hsp90) upon chronic oxidative stress (Figure 7r). Likewise, Interleukin (IL)‐13 and transforming growth factor (TGF)‐β, which are involved in mechanisms of inflammation and immune response (Fu et al., 2017; Kliffen et al., 1997), also showed increased expression in both ipRPE cells (IL‐13) and ipRPE‐EVs (IL‐13 and (TGF)‐β) following chronic oxidative stress (Figure 7s). Most notably, increased expression of several drusen associated proteins known to be strongly linked to the progression of AMD was observed in chronic oxidative stress conditions in ipRPE cells (ApoE, VTN, C3 and VIN) and ipRPE‐EVs (ApoE, VTN, VIN and CLU) (Figure 7t and S7).

Thus, these results provide first evidence that drusen proteins are released as cargo of EVs secreted by RPE cells and, that these proteins are enriched in RPE‐EVs under chronic oxidative stress conditions.

### 3.6 ipRPE‐EVs display directional release of drusen‐associated proteins in homeostatic conditions and this directionality is modulated in response to chronic oxidative stress

Having demonstrated that ipRPE monolayers exhibit distinct apical and basolateral EV proteomes (Figure 6a; protein list in Table S2b and c) as well as increased EV release in response to chronic oxidative stress (Figure 7p), we evaluated whether ipRPE‐EVs exhibit directional differences in drusen‐associated protein cargo under homeostatic conditions, and whether this directional release is susceptible to modulation in response to a stressor relevant to AMD such as chronic oxidative stress.

**FIGURE 8:**
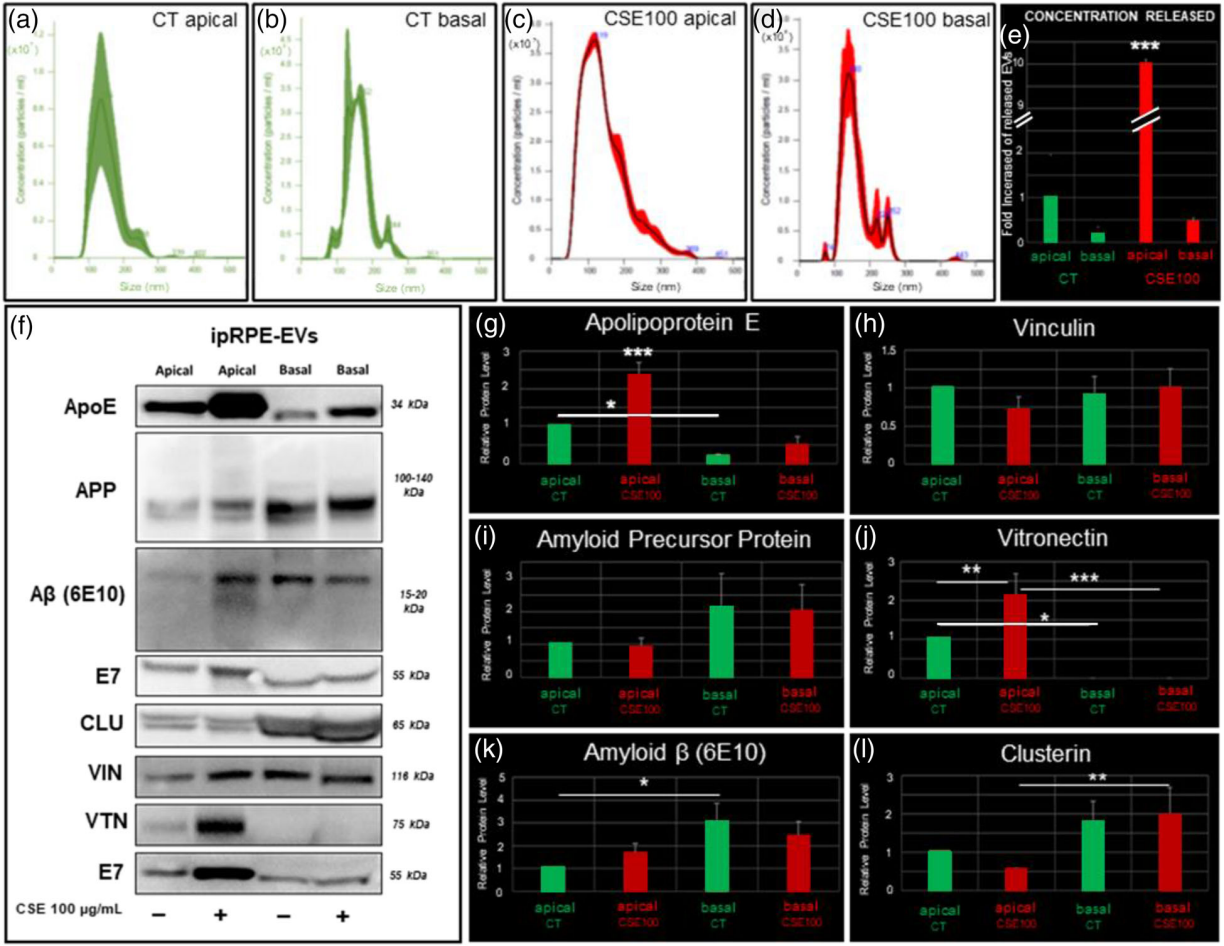
Directional release of drusen‐associated proteins is modulated in response to chronic oxidative stress. Nanoparticle tracking analysis revealed the size distribution of released EVs by control ipRPE cells (apical (a) and basal (b) side), and by CSE‐treated ipRPE cells (apical (c) and basal (d) side). CSE‐treated ipRPE cells displayed increased release of EVs compared to non‐treated ipRPE (e). Drusen‐associated proteins present in EVs from ipRPE monolayers under CSE or normal conditions were confirmed by Western Blot (f) and analyzed by quantitative western blot analysis (*n* = 3) (g–l). Bar graphs represent mean ± SD. *p < 0.05; **p < 0.005; ***p < 0.0005. *n* = 3

Consistently with our observations of an overall increase in ipRPE‐EV secretion following chronic oxidative stress (Figure 7p), increased ipRPE‐EV release was observed from both apical and basal sides under these conditions. Notably, this increase in EV release was not bidirectionally proportional, but rather preferentially shifted towards the apical side, with a 10‐fold apical increase compared to 4‐fold basal increase (Figure 8a–e). Importantly, several drusen‐associated proteins concomitantly exhibited increased release in ipRPE‐EVs from both apical and basal sides compared to homeostatic conditions (Figure 8f–l). Iodixanol density gradient conclusively demonstrated that these drusen‐associated proteins are released in association with ipRPE‐EVs (Figure S8).

Remarkably, quantitative western blot analysis for these selected drusen‐associated proteins demonstrated both, distinctive directional secretion modes in homeostatic conditions and, differential modulation of this directional secretion in response to chronic oxidative stress (Figure 8g–l). Under homeostatic conditions, some drusen‐associated proteins exhibited proportional release to both apical and basal sides (VIN; Figure 8h), while others showed apical > basal (ApoE, VTN; Figures 8g and j) or apical < basal (APP, Aβ, CLU; Figures 8i, k and l) directional mode. Of note, VTN was only detected in EVs apically secreted (Figure 8j). Notably these apical:basal directional modes were differentially modulated upon chronic oxidative stress. A 2‐fold increase in ApoE release within EVs from both the apical and basal sides was observed (Figure 8g) and the apical:basal ratio was maintained between the two conditions (5:1). Similarly, the apical:basal ratio for APP was also maintained between the two conditions (1:2), though no fold‐changes were observed for either apical or basal release (Figure 8I). On the other hand, while Aβ still showed an apical < basal mode comparable to homeostatic conditions (Figure 8k), a shift on the apical:basal ratio favouring apical directional secretion was observed under chronic oxidative stress (apical:basal ratio 1:3 in homeostatic conditions vs. 2:3 under chronic oxidative stress). In contrast, VIN and CLU exhibited a shift on the apical:basal ratio favouring basal directional secretion following chronic oxidative stress compared to homeostatic conditions, with respective apical:basal ratios of 1:1 versus 2:3 for VIN and 1:2 versus 1:3 for CLU (Figure 8h and l). Finally, VTN showed exclusive apical directional release in both, homeostatic and chronic oxidative conditions with a 2‐fold enrichment under chronic oxidative stress. (Figure 8j). Of note, the overall increased secretion for all these proteins to both apical and basal sides upon chronic oxidative stress is significantly greater than their fold‐enrichment. Enrichment, overall increased secretion, and apical:basal mode for these drusen‐associated proteins in response to chronic oxidative stress are summarised in Table S3.

These observations strongly support the existence of a specialised mechanism regulating directional apical:basal sorting and secretion of drusen‐associated proteins via EVs, which is finely modulated in response to mechanisms involved in AMD pathophysiology.

## 4 DISCUSSION

We have established a simple and efficient strategy to derive functionally mature polarised RPE monolayers analogue to human primary RPE directly from the RPE tissue associated to human retinal organoids. Moreover, we have isolated and characterised EVs secreted from both, the apical and basolateral surfaces from our human induced primary RPE (ipRPE) system and profiled their proteomic cargo. These studies revealed that ipRPE‐derived EVs were selectively enriched in proteins involved in pathways associated to oxidative stress, immune response, inflammation, complement system and, notably, drusen composition, all mechanisms participating in AMD pathophysiology. Furthermore, using our ipRPE system we established a model that combines several key aspects of AMD (including aeging, chronic oxidative stress induced by cigarette smoke exposure and drusen‐like deposits) and applied this experimental platform to the analysis of the dynamics of RPE‐EV secretion and protein cargo. Remarkably, EVs secreted by RPE cells within an AMD‐like environment significantly enhanced the release of AMD‐associate proteins ApoE, Aβ, CLU, VIN and VTN, all crucial proteins in drusen formation (Crabb et al., 2002). Taken together these results strongly support a significant role of EVs in the physiology of the retina and its potential involvement in the pathophysiology of AMD. Most notably, we have demonstrated that proteins linked to drusen formation are released in association with EVs, providing first evidence for a possible active role of RPE‐derived EVs in drusen development and growth.

During the last decade, there has been significant interest in the generation of in vitro RPE systems to study retinal disease mechanisms and to deliver substrates for potential cell‐based therapies (Canto‐Soler et al., 2016). Most protocols to derive human RPE monolayers from stem cells require enrichment and sub‐culturing of pigmented colonies and/or free‐floating aggregates, and a regime of exogenous factors to induce stepwise differentiation (Table S4). In contrast, we have established a straightforward method to derive polarised functionally mature human RPE monolayers directly from hRetOs by mimicking the approach to derive cultures of primary human RPE cells (Blaauwgeers et al., 1999; Geisen et al., 2006; Hu & Bok, 2001; Maminishkis et al., 2006; Sonoda et al., 2009). Having validated that the RPE tissue associated to our hRetOs is analogous to the native human RPE in its timing of differentiation, histological organisation, and key features of functional maturation, we further demonstrate that RPE derived monolayers emulate the behaviour of primary human RPE cultures and exhibit functional hallmarks of bona fide mature RPE cells, including proper ultrastructural differentiation, expression of genes involved in essential mechanisms of RPE maturation and function, active phagocytosis of photoreceptor outer segments and polarised secretion of bioactive molecules, all as in the native tissue and its derived primary cultures. Importantly, we further demonstrate the suitability of our ipRPE system to mimic the biology and function of the native human RPE by its ability to recreate molecular and phenotypic hallmarks of early AMD pathogenesis, including drusen‐like deposits, when exposed to environmental stressors known to contribute to AMD onset and progression.

The RPE forms a tight‐junction epithelium located between the blood flow of the choroid and the outer segments of the photoreceptors (Strauss, 2005) and plays a critical role in maintaining the homeostasis between these three tissues (Fuhrmann et al., 2014). To communicate with its neighbouring tissues, the RPE secretes a large variety of factors and signalling molecules. EVs are increasingly being recognised as key players regulating cell–cell and tissue–tissue communication (Colombo et al., 2014), and even though our understanding of the biology, function and translational potential of EVs is rapidly increasing (Ratajczak & Ratajczak, 2020; Dai et al., 2020; Hill, 2019), knowledge in the eye field is still in the early stages (Klingeborn et al., 2017). The majority of previous studies on human EVs secreted from RPE cells (Table S5) have used ARPE‐19 cells (Atienzar‐Aroca et al., 2016; Biasutto et al., 2013; Gangalum et al., 2011; Gangalum et al., 2016; Knickelbein et al., 2016; Mckechnie et al., 2003; Wang et al., 2009), which are deficient in essential hallmarks of bona fide RPE (Beebe, 2013). Of the remaining studies, only three have been carried out in human fetal (Sreekumar et al., 2010), human donor eyes (Locke et al.^2014^) or hiPS cells (Singh et al., 2015). Most notably, proteomic analysis of RPE‐EV cargo has been performed in only two cases, in EVs derived from ARPE‐19 (Biasutto et al. 2013) and porcine primary RPE cultures (Klingeborn et al., 2017). Thus, our study is the first to isolate EVs from human RPE, characterise its proteome cargo, and analyse the dynamics of EV secretion and proteome from the RPE apical and basal side, in both homeostatic and AMD‐like environments.

The physical characteristics and biochemical composition of our ipRPE‐EV preparations fulfill the criteria for EVs containing exosomes and microvesicles (Théry et al., 2018). Since both, exosomes and microvesicles, are equally emerging as integrators of homeostasis with important roles in physiological and pathological states (Stahl & Raposo, 2019), and since they are normally released as a mixed poplulation, we though best to conduct our study as a comprehensive analysis of the EV pool. Accordingly, our proteomic analyses revealed that ipRPE‐EVs carry proteins involved in key RPE biological processes, tissue homeostasis, and mechanisms of retinal development, as well as pathways associated to neurodegenerative diseases (Table S1). Of note, 3% of human proteins identified in ipRPE‐EVs have not been previously documented to exist in EVs.

EVs released from ipRPE monolayers under homeostatic conditions were selectively enriched in proteins associated to oxidative stress, immune response, inflammation, complement system and drusen composition, all mechanisms involved in AMD pathophysiology. In particular, processes such as hypoxia, cellular response to ROS and oxidative stress, regulation of defense response, response to heat shock and aeging, cellular senescence and antioxidant activity, all point to a potential role of EVs in cellular response to oxidative damage. The identified enriched proteins are also linked to pathways related to humoral and adaptive immune response, immune effector process, innate immune system, activation of immune response, immunoglobulin complex (MHC), regulation of macrophage activation and regulation of cytokine production involved in immune response and immunoglobulin expression, thus suggesting EV cargo may also play a role in modulating immune response in distant recipient cells. In addition, pathways normally triggered during inflammatory process were also linked to proteins found in ipRPE‐EVs, such as cytokine production and secretion, regulation of Inflammatory response, regulation of TNF superfamily cytokine production, NLRP3 inflammasome and production and signalling of interleukins 1, 2, 4, 6, 8,12 and 13.

Notably, ipRPE monolayers exhibited distinct apical and basolateral EV proteomes, supporting a directional enrichment mechanism of protein sorting and secretion in RPE tissue via EVs, through which specific EV cargo would be delivered toward the Bruch membrane, choroid, and systemic circulation or into the subretinal space. Of special relevance, ipRPE‐EVs contained drusen‐associated proteins, which exhibited differential apical:basal directional release, with some of them showing proportional release to both apical and basal sides, while others show apical > basal or apical < basal directional mode. Most notably these apical:basal directional modes were differentially modulated upon chronic oxidative stress. These results provide first evidence that drusen proteins are released in association with EVs secreted by RPE cells and uncover a response mechanism from RPE to AMD stressors via exosomes and microvesicles.

Following chronic oxidative stress, ipRPE responded with a dramatic increase in EV secretion from both apical and basal sides. These observations highlight the bioavailability of EVs in response to stress‐related changes and correlate with previous studies showing upregulation of EV markers and increased EV release in ARPE‐19 when subjected to oxidative damage (Wang et al., 2009; Shah et al., 2018) or inflammatory cytokines (Knickelbein et al., 2016), as well as those from Singh and colleagues showing bi‐directional increase of EV release in hiPSC‐RPE from Best disease patients (Singh et al., 2015). Interestingly, in our studies the increase in EV release was preferentially shifted towards the apical side, suggesting the involvement of a highly controlled directional mechanism of EV secretion. Unexpectedly however, even though drusen‐associated proteins exhibited a concomitant increased release in ipRPE‐EVs, this increase was not proportionally correlated with the apical > basal shift observed in EV secretion, but rather differentially modulated for each protein. Although we cannot fully rule out the possibility of EVs being trapped within the transwell pores, in light of the recent observations from Klingeborn et al. (2017), this effect is unlikely to fully account for the differences seen in our study in apical versus basal EV release.

Despite our growing understanding of AMD (Ambati & Fowler, 2012; Bhutto & Lutty, 2012; Bok, 2005; Green, 1999; Mullins et al., 2000) little is known about the origin of drusen‐associated proteins (Crabb et al., 2002; Hageman, 2001). While some studies indicated that drusen proteins are mainly derived from cellular debris from processed photoreceptor outer segments and the RPE, others suggest a choroidal cell or blood origin (Bergen et al., 2019). Interestingly, a decade ago Wang and colleagues hypothesised that the release of intracellular proteins via EVs by the aged RPE might contribute to the formation of drusen (Wang et al., 2009). Thus, it has been suggested that in the aged RPE in vivo, increased exocytotic activity leads to the release of intracellular proteins via EVs which potentially contribute to the formation of drusen (Klingeborn et al., 2017; Lakkaraju et al., 2020; Wang et al., 2009). Here we provide first evidence of well‐known drusen proteins being released in association with EVs, and of a finely‐tuned mechanism of drusen‐associated protein sorting in response to stressors relevant to AMD. Collectively, these results strongly support an active role of RPE‐derived EVs as a key source of drusen‐associated proteins and important contributor to drusen development and growth.

We postulate that, RPE increased release of drusen proteins via EVs in response to AMD stressors such as chronic oxidative stress may initially represent a protective mechanism, since proteins found in drusen are known to play important roles as anti‐angiogenic (Browning et al., 1994), anti‐inflammatory (Kelly et al., 1994; Mclaughlin et al., 2000, Wasmuth et al., 2009) and anti‐oxidative factors (Kim et al., 2010; Tangirala et al., 2001), as well as neuroprotective of RPE and photoreceptor cells (Vargas et al., 2017). In turn, excessive production and secretion of these proteins, would lead to the spread of the toxic forms of proteins (Alvarez‐Erviti et al., 2011; Sardar Sinha et al., 2018), propagation of RPE disfunction (Anderson et al., 2002; Datta et al., 2017; Sakaguchi et al., 2002), and formation of drusen deposits (Anderson et al., 2004; Bergen et al., 2019; Hageman et al., 1999; Sakaguchi et al., 2002). The well documented interplay between ApoE and Aβ supports this possible scenario. ApoE plays an essential protective role in neuronal response to injury as an anti‐angiogenic (Browning et al., 1994), anti‐inflammatory (Kelly et al., 1994) and anti‐oxidative factor (Tangirala et al., 2001), while it is also known to regulate Aβ metabolism, aggregation and deposition (Kanekiyo et al., 2014). In Alzheimer's disease, ApoE downregulates exosome biosynthesis and secretion (Peng et al. 2019) and stimulates Aβ production leading to Aβ extracellular deposition (Anderson et al., 2004; Anderson et al., 2001; Riva et al., 2019; Yoshida, 2005). In addition, when Aβ concentration overwhelms the lysosomal degradation compartment leads to Aβ aggregation (Hu et al., 2009; Li et al., 2012) and propagation of toxic Aβ aggregates via EVs (Riva et al., 2019; Sardar Sinha et al., 2018). Similarly, apical increased release of VTN and ApoE may represent another scenario of this protective versus pathologic balance. Enhanced VTN might represent a protective mechanism in photoreceptor cells against complement activation by inhibiting the formation of the membrane attack complex (Katschke et al., 2018; Lueck et al., 2011; Su, 1996), while excessive ApoE release may lead to the induction of inflammatory cytokines promoting subretinal inflammation (Levy et al., 2015).

In addition to the selective increased release of drusen‐associated proteins under chronic oxidative stress, ipRPE‐EVs also showed increased secretion of proteins involved in cellular response to oxidative stress, inflammation and immune response, all well‐known key mechanisms of RPE stress in AMD and other retinal diseases (Anderson et al., 2002; Beatty et al., 2000; Faber & Nissen, 2008; Jha et al., 2006; Nita & Grzybowski, 2016; Whitcup et al., 2013). Specifically, increased release of SOD1, Hsp90, TXN, IL‐13 and TGFb suggest an important potential role of RPE derived EVs in protecting both, neighboring RPE and photoreceptor cells, from disease mechanisms such as ROS‐mediated damage (Behndig et al., 1998; Sugano et al., 2013), inflammation (Gimeno‐Hernández et al., 2020) and protein aggregation (Lamark & Johansen, 2012). Thus, EVs may represent a mechanism by which RPE maintains a balanced homeostatic environment to protect neighbouring RPE cells as well as adjacent endothelial cells and photoreceptors from inflammatory and oxidative damage. On the other hand, it is known that EVs play a role in spreading the toxic forms of proteins in other neurodegenerative diseases (Rajendran et al., 2006), and thus it is also plausible that RPE‐derived EVs may contribute to the mechanisms of propagation of RPE dysfunction in AMD.

In summary, our studies provide a novel method for deriving functionally mature RPE monolayers, modelling of AMD, and the first evidence that drusen proteins are released in association with EVs secreted by RPE cells through a finely‐tuned apical:basal sorting mechanism, which is modulated in response to AMD‐linked stressors. Collectively, our results strongly support an active role of RPE‐derived EVs as key source of drusen‐associated proteins and important contributors to drusen development and growth.

## AUTHOR CONTRIBUTIONS

Miguel Flores‐Bellver, M. Valeria Canto‐Soler and Stephen Redenti designed research. Miguel Flores‐Bellver, Jason Mighty, Silvia Aparicio‐Domingo, Kang V. Li, Cui Shi, Hannah Cobb, Jing Zhou, Patrick McGrath, German Michelis, Patricia Lenhart, Michael J. Rudy, S. Patricia Becerra, Ganna Bilousova, Søren Heissel, Christina Coughlan, Andrew E. Goodspeed, Stephen Redenti and M. Valeria Canto‐Soler are responsible for research execution and are contributors to data acquisition. Miguel Flores‐Bellver, Stephen Redenti and M. Valeria Canto‐Soler are the primary contributors to data analysis and interpretation. Manuscript preparation by Miguel Flores‐Bellver and M. Valeria Canto‐Soler with revisions provided by Stephen Redenti and S. Patricia Becerra.

## Supporting information

Supporting Information

Supporting Information

Supporting Information

Supporting Information

Supporting Information

Supporting Information

Supporting Information

TableS1

TableS2

TableS4

## Data Availability

All data needed to evaluate the conclusions in this study are presented in the main text and/or Supplementary Materials.
