# Supplementary material for: Extracellular vesicles released by human retinal pigment epithelium mediate increased polarised secretion of drusen proteins in response to AMD stressors
Source: J Extracell Vesicles. 2021 Nov 8;10(13):e12165. doi: 10.1002/jev2.12165 (PMC8575963; doi:10.1002/jev2.12165)
Supplement: Supplementary file 1 — Supporting Information [file JEV2-10-e12165-s001.docx]

**Extracellular vesicles released by human retinal pigment epithelium mediate increased polarized secretion of drusen proteins in response to AMD stressors**

**Short title: Polarized secretion of drusen-associated proteins via extracellular vesicles**

M. Flores-Bellver ^1*^, J. Mighty ^2, 3^, S. Aparicio-Domingo ^1^, K. Li ^1^, C. Shi ^2, 3^, H. Cobb ^1^, J. Zhou ^2^, P. McGrath ^4^, G. Michelis ^5^, P. Lenhart ^1^, S. P. Becerra ^5^, G. Bilousova ^4,6,7^, S. Heissel ^8^, M. J. Rudy ^13^, C. Coughlan ^9^, A.E. Goodspeed ^10,11^, S. Redenti ^2, 3, 12^, M.V. Canto-Soler ^1, 6*^.

1. *CellSight* Ocular Stem Cell and Regeneration Program, Department of Ophthalmology, Sue Anschutz-Rodgers Eye Center, University of Colorado, School of Medicine, Aurora, CO, USA.

2. Lehman College, Bronx, NY, USA.

3. Biology Doctoral Program, The Graduate School and University Center, City University of New York, New York, NY, USA.

4. Department of Dermatology, University of Colorado School of Medicine, Anschutz Medical Campus, Aurora, CO, USA.

5. Section of Protein Structure and Function, NEI, NIH, Bethesda, MD, USA.

6. Charles C. Gates Center for Regenerative Medicine, University of Colorado School of Medicine, Anschutz Medical Campus, Aurora, CO, USA.

7. Linda Crnic Institute for Down Syndrome, University of Colorado School of Medicine, Anschutz Medical Campus, Aurora, CO, USA.

8. Proteomics Resource Center, The Rockefeller University, New York, NY, USA.

9. University of Colorado Alzheimer’s and Cognition Center, Department of Neurology, Linda Crnic Institute for Down Syndrome, University of Colorado Anschutz Medical Campus, Aurora, CO, USA.

10. Department of Pharmacology, University of Colorado, Aurora, CO, USA.

11. University of Colorado Cancer Center, Aurora, CO, USA.

12. Biochemistry Doctoral Program, The Graduate School, City University of New York, New York, NY, USA.

13. Department of Neurology, University of Colorado School of Medicine, Aurora, CO 80045, USA.

^*^ Co-corresponding authors: m.flores-bellver@cuanschutz.edu, valeria.canto-soler@cuanschutz.edu.

**Supplementary Materials**

**Figure S1. Isolation and culture of human induced pluripotent stem cells-derived RPE.** Characteristic hRetOs with a transparent pseudostratified neuroretina epithelium continuous with the adjacent pigmented RPE (**A,** white asterisks) on D25 and D35 (**B**). The RPE domain acquired a spheroid shape with variable size (**C** and **C’**) and became pigmented (**C-D**). Cellular morphology of passaged and expanded RPE monolayers on transwell inserts (**E**) and, in regular plastic plates (**F** and **G**); when grown in plastic plates RPE monolayers displayed formation of fluid-filled domes reflective of polarized functional properties (**F**-**G**). Scale bars, 100 μm.

**
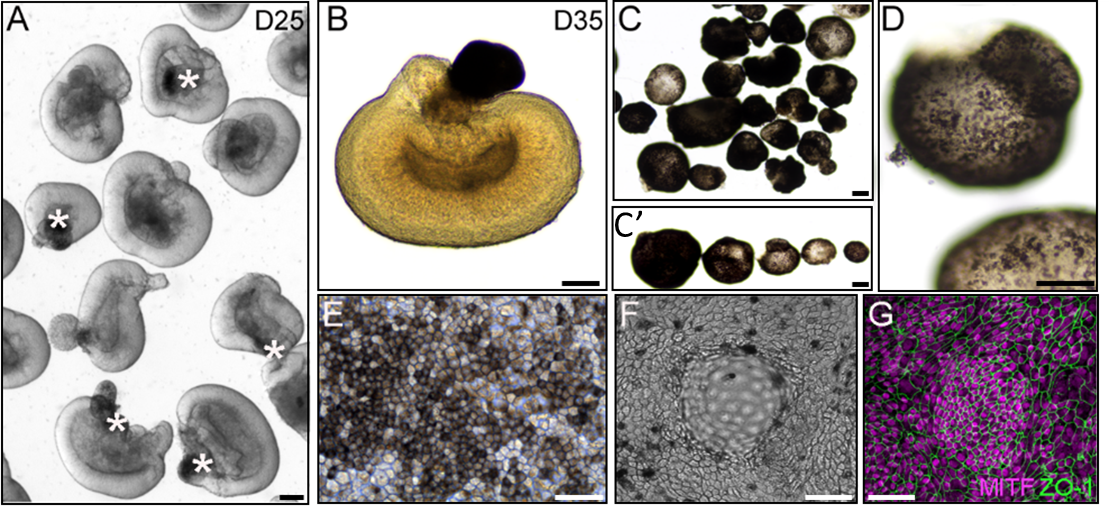
**

**Figure S2. Human iPSC lines used in this study**. Three human iPSC lines of different cell origin and reprogramming method were chosen to test the reproducibility of our method across cell lines (**A**). Expression of key pluripotency markers by immunostaining, after 5 days of culture (**B**). The three different hiPSC lines demonstrated consistent and reproducible derivation of RPE monolayer cultures (**B**), including the expression of key functional genes by RT-PCR (**C**). Scale bars, 100 μm.


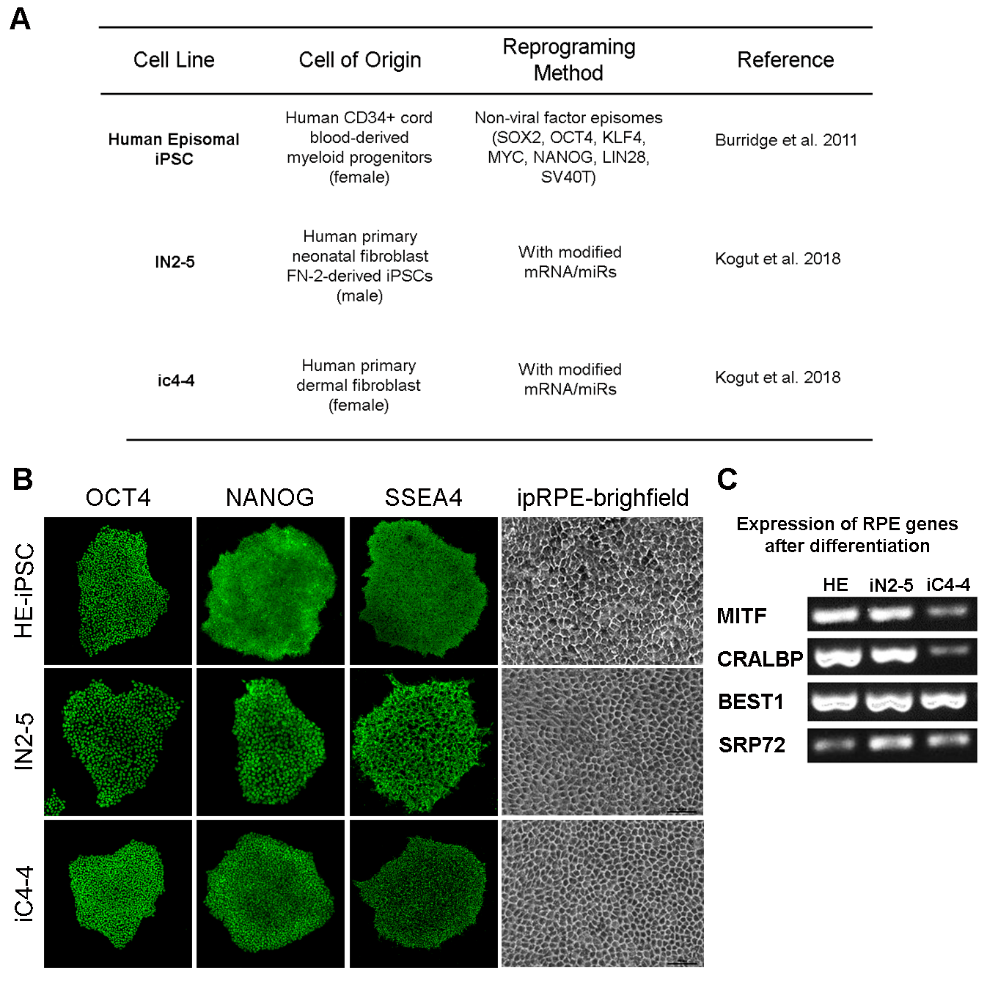


**Figure S3. Characterization of human induced-primary RPE (ipRPE) monolayers from passage 1 to passage 4**. Schematic displaying the protocol used to passage and expand ipRPE cultures (**A**). RT-PCR analysis of key RPE markers expressed by P1-P4 ipRPE monolayers (**B**). Western blot analysis showing protein expression of RPE markers throughout sequential subculture of ipRPE monolayers (**C**). Immunostaining of MITF (green) and ZO-1 (purple) revealed areas of confluent RPE cells interrupted by acellular areas in P4 ipRPE (**D**). Quantitative PCR comparing level of expression of key RPE markers in P1 and P2 ipRPE after 50 days of maturation (**E**). Transepithelial resistance (TER) levels of polarized ipRPE monolayers comparing P1 and P2 (**F**); red dotted line represents the TER of human RPE in vivo^1^. Polarized secretion of VEGF-A (**G**) observed in ipRPE (P1 and P2). Capability of the ipRPE monolayers to expand after sequential subculture (P1 and P2) (**H**). Bar graphs represent mean ± SD. *p < 0.05; **p < 0.005; ***p < 0.0005. Scale bar, 50 μm. n=3.


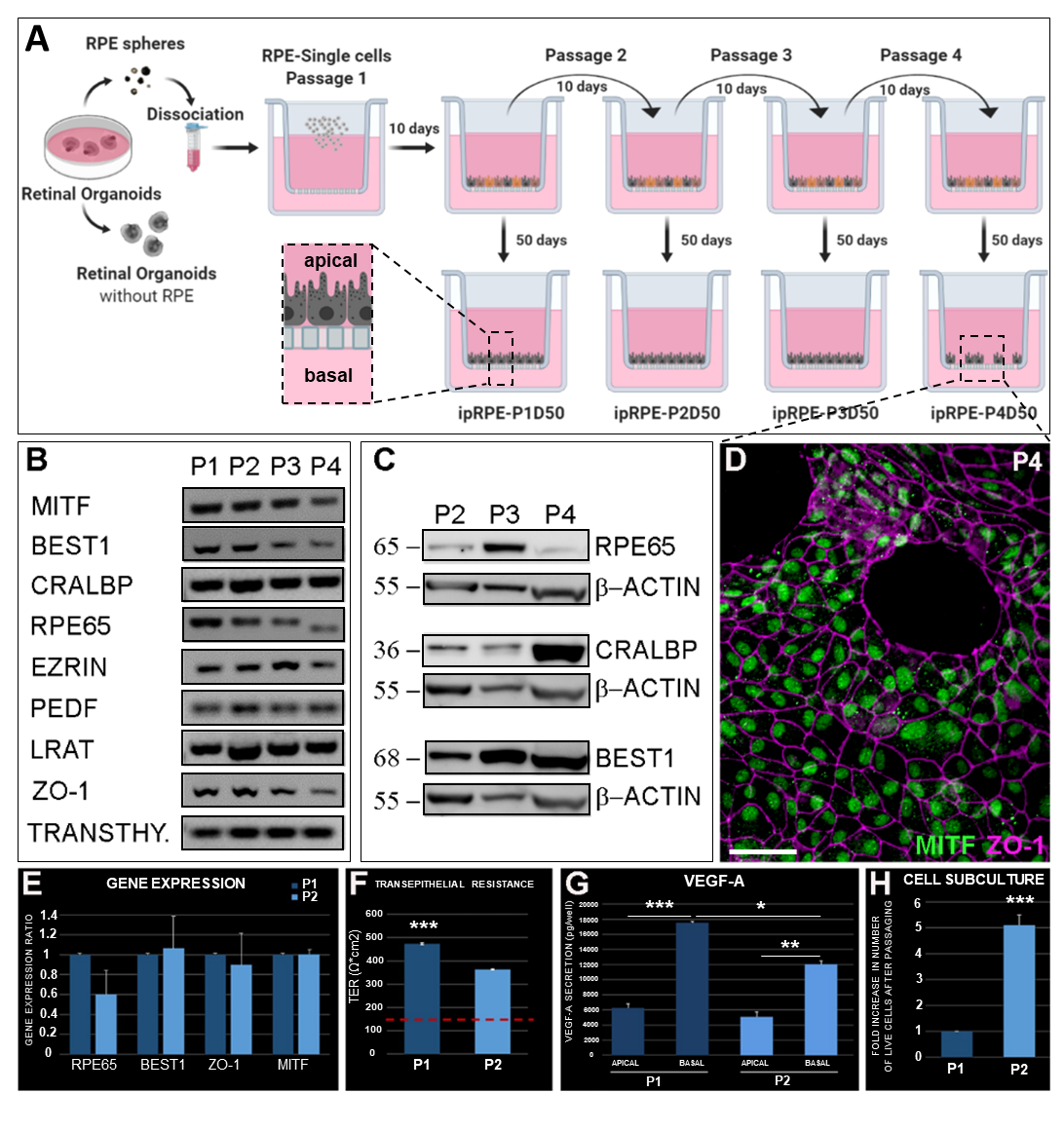


**Figure S4.** **ipRPE-derived extracellular vesicles (EVs) during development.** EVs were isolated from either apical or basal conditioned media at different time points during ipRPE development. Nanoparticle tracking analysis (NTA) revealed the size distribution for apical (**A**) and basal (**B**) sides on day 15 (D15) of ipRPE maturation, the average concentration (**C**) and the average size of released EVs (**D**). EVs secreted by ipRPE on day 30 (D30) via apical (**E**) and basal sides (**F**) were analyzed by NTA, including their concentration release (**G**) and average size (**H**). Tables indicate the concertation of released EVs (**I**) and their size (mode and mean) (**J**) at different stages of maturation (15, 30 and 50 days). Bar graphs represent mean ± SD. **p < 0.005; ***p < 0.0005. n=3.


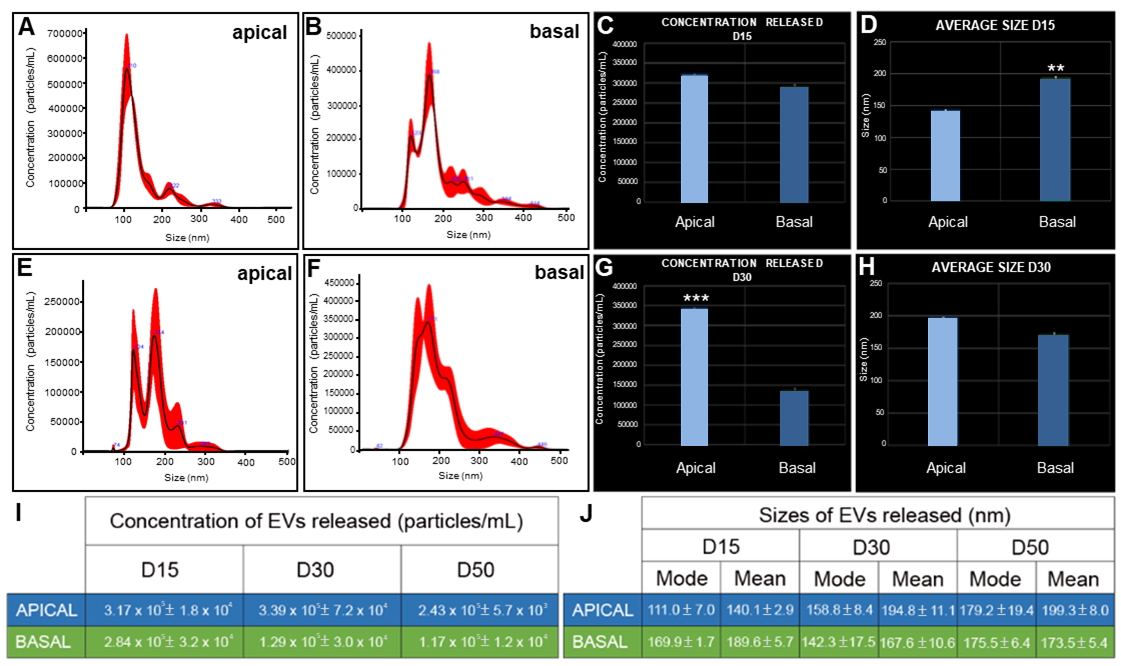


**Figure S5. Biological processes of induced-primary RPE-derived EVs.** Bioinformatic analysis was performed to classify EV protein cargo into predicted biological processes for apical ipRPE-EVs (**A**), basal ipRPE-EVs (**B**), and those exclusive to the apical (**C**) or the basal (**D**) released EVs.


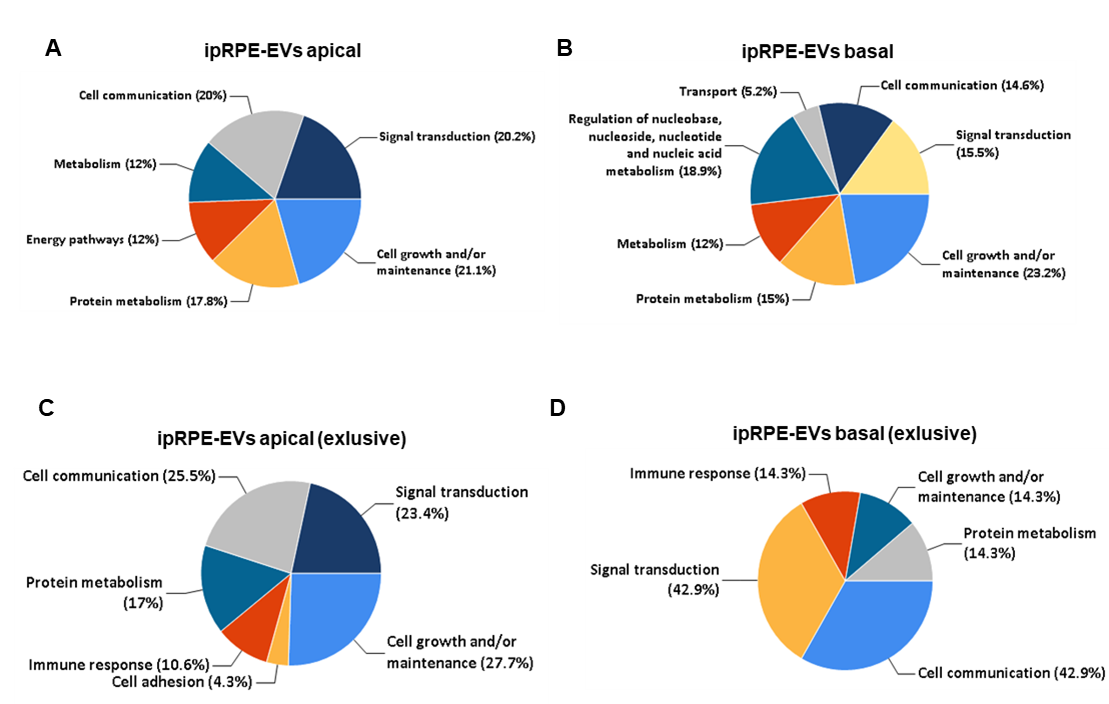


**Figure S6.** **Acute exposure to cigarette smoke extract (CSE) induces cell death and oxidative stress in ipRPE monolayers.** Representative fluorescence photomicrographs of phalloidin (green) and TUNEL positive (purple)-stained ipRPE cells in untreated controls (**A**) and cells treated with 50 (**B**), 100 (**C**) and 200 (**D**) ug/mL of CSE for 24 hours**.** Quantification of the number of non-viable cells (TUNEL positive) after acute CSE exposure (**E**). ROS production in CSE-treated ipRPE monolayers occurred in a concentration-dependent manner (**F**). Bar graphs represent mean ± SD. *p < 0.05; ***p < 0.0005. Scale bars, 50 μm (**A**–**D**). n=3.


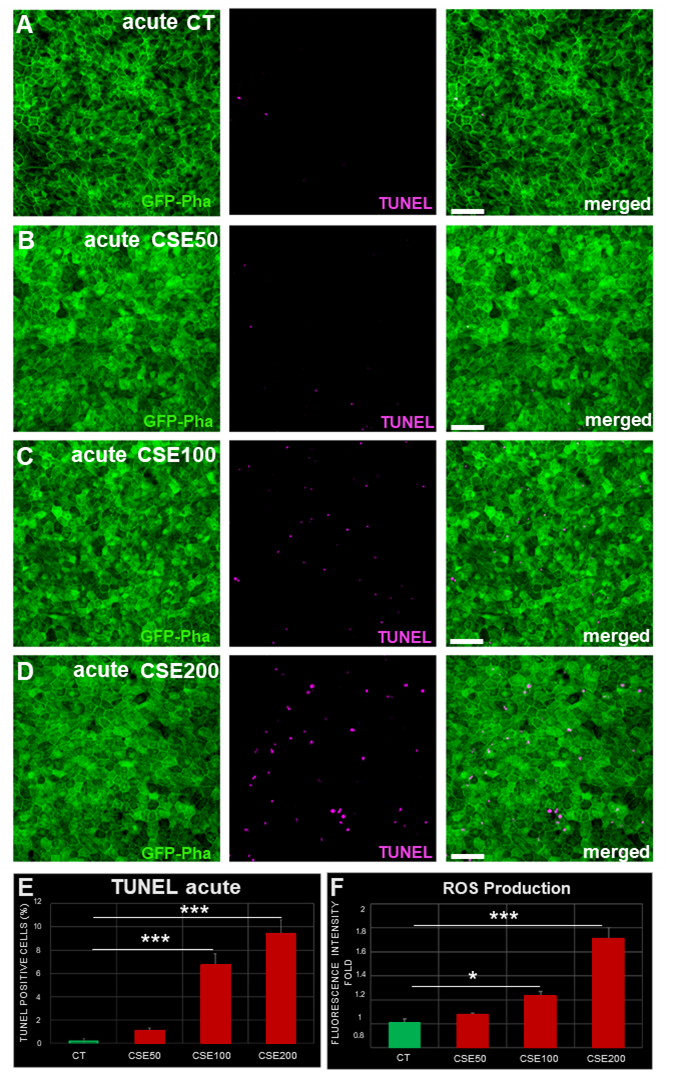


**Figure S7. Drusen-associated protein expression is modulated in response to chronic oxidative stress in ipRPE cells.** Quantification of western blot was performed in cell lysates from non-treated ipRPE cells and CSE-treated ipRPE cells (**A**): ApoE (**B**), APP (**C**), Aβ (**D**), VIN (**E**), VTN (**F**) and CLU (**G**). Bar graphs represent mean ± SD. *p < 0.05; **p < 0.005; ***p < 0.0005. n=3.


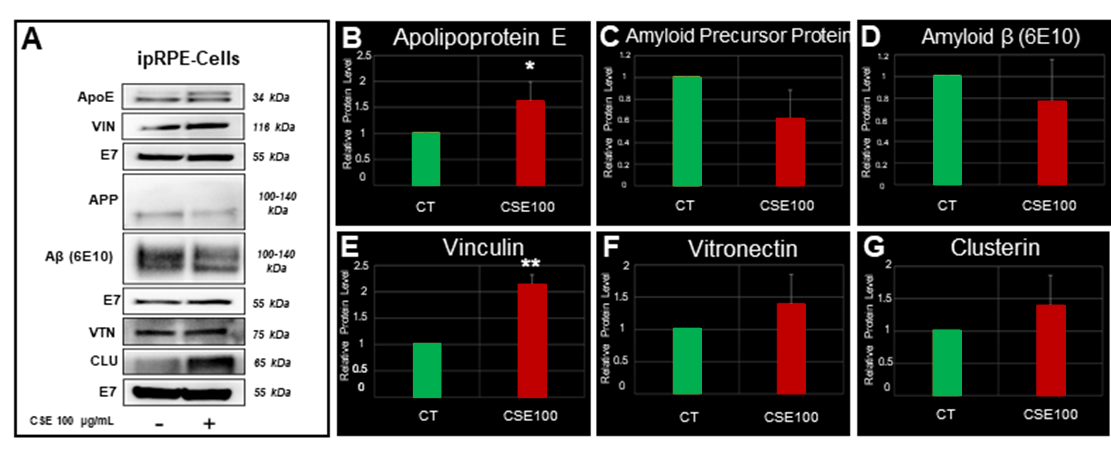


Please, see attached files related to: **Table S1. Enrichment pathways analysis of** **ipRPE-EVs;** and **Table S2. Proteome profile, row data (S2a, S2b and S2c).**

**Table S3. Enrichment, overall increased secretion, and apical:basal mode of drusen-associated proteins secreted via EVs in response to AMD stressors.** Enrichment level represents the fold-increase protein expression in EVs from chronic oxidative stress relative to homeostatic conditions. The overall increase in secretion was calculated based on the enrichment level and fold-increased release of EVs relative to homeostatic conditions. The apical:basal mode represents the apical:basal ratio for homeostatic and CSE conditions. A: apical; B: basal

**TABLE S4. Human stem cell-derived RPE systems.**

*Please see attached supplementary file.*

**Figure S8. Drusen proteins are associated with EV fractions.** (A) Iodixanol density gradient tube after 60 h ultracentrifugation. (B) Representative immunoblots of iodixanol density gradient fractions were performed with antibody against CD63, a canonical exosome marker. Densities of fractions 5, 6 and 7 correspond to the density of exosomes. Drusen associated proteins (APP, ApoE, Ab(6E10), VTN and VIN) were mainly detected in exosome fractions.

*
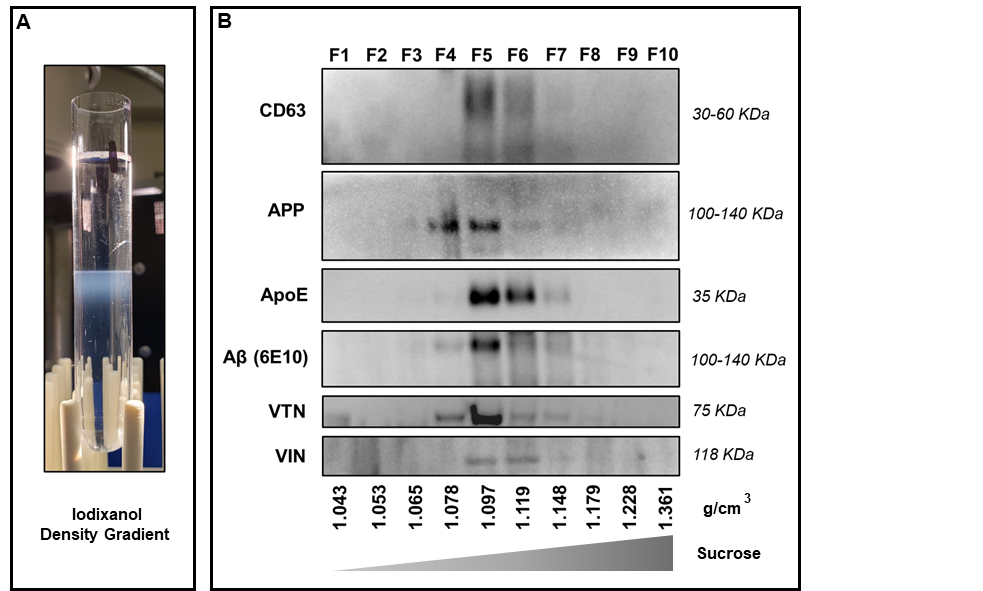
*

**Table S5.** **Studies to date on isolation and characterization of RPE-derived extracellular vesicles.**

| **Author** | **Cell type** | **Method** | **Chamber** | **Inc. (h)** | **TEM** | **WB** | **qPCR/RNA** | **FACS/ELISA** | **Nano-Sight** | **Prot.** | **Co-culture** | **Key Proteins** |
| --- | --- | --- | --- | --- | --- | --- | --- | --- | --- | --- | --- | --- |
| **McKechnie et al. 2003 ^2^** | ARPE-19 | UC | F | 48 | X | X |  |  |  |  |  | CD63; Hr44 |
| **Wang et al. 2009 ^3^** | ARPE-19 | UC | R | N/A |  |  |  | X |  |  |  | CD63; CD81;  CFH; C3; LAMP2 |
| **Sreekumar et al 2010 ^4^** | hfRPE | UC | F/TW | 24 | X | X |  |  |  |  |  | CD63; CRYAB |
| **Gangalum et al 2011 ^5^** | ARPE-19 | UC | R/TW | N/A | X |  |  |  |  |  |  | CRYAB; Hsp70;  flot-1; ENO1 |
| **Toyofuku et al 2012 ^6^** | mRPE/ARPE-19 | UC/EQ | TW | 48 |  | X |  |  |  |  |  | CRYAB; Hsp70;  Tsg101; PSAP; pCD; SEMA4A |
| **Biasutto et al 2013 ^7^** | ARPE-19 | UC | F | 24 |  |  |  |  |  | X |  | CD63; CD9; RPE-65;  TRL3; VEGFR2; PDGFRβ |
| **Locke et al. 2014 ^8^** | hRPE**^†^** | UC | N/A | 0.017 |  | X |  |  | X |  |  | MYOC |
| **Aboul Naga et al. 2014 ^9^** | pRPE | UC | N/A | 24 | X | X |  | X |  |  |  | CD63; Tsg101 |
| **Singh et al. 2015 ^10^** | hiPSC-RPE | UC | TW | 24 |  | X |  |  | X |  |  | CD63 |
| **Knickelbein et al 2016 ^11^** | ARPE-19 | EQ | F | 48 |  | X |  | X | X |  | WBCs | CD81; CD63 |
| **Gangalum et al. 2016 ^12^** | ARPE-19 | UC | TW | 12-14 | X | X |  |  |  |  |  | CD63; CRYAB; Hsp70;  Hsp60; flot-1; ENO1; Alix |
| **Atienzar-Aroca et al 2016 ^13^** | ARPE-19 | UC | R | 24 | X | X | X | X* |  |  | HUVEC | CD9; VEGFR1; VEGFR1 |
| **Klingeborn et al 2017 ^14^** | pRPE | UC/G | TW | 24 | X | X |  |  | X | X |  | CD63; CD81; CD9; EZR;  BEST1; RLBP1; CRYAB;  SDCBP; SLC39A12 |
| **Shah et al 2018 ^15^** | pRPE/ARPE-19 | EQ | TW | 72 | X |  |  | X | X |  | ARPE-19 | No proteins |
| **Atienzar-Aroca et al 2018 ^16^** | ARPE-19 | UC | R | 24 | X |  |  | X |  |  | HUVEC | CD9; BAX; Bcl2; Atg12 |
| **Maisto et al 2019 ^17^** | ARPE-19 | UC | R | 24 | X |  |  | X* | X |  | HUVEC | CD9; VEGF; CD81 |
| **Oltra et al 2019 ^18^** | ARPE-19 | UC/EQ | R | 24 | X |  | X |  | X |  |  | No proteins |
| **Zhang et al 2019 ^19^** | ARPE-19 | UC | R | 24 | X | X |  |  |  |  |  | CD9; CD81; CD63; IL-8; IL-1β; Caspase 1 |
| **Ferreira et al 2019 ^20^** | ARPE-19 | UC/G | R | 24 | X | X |  |  | X |  |  | CD63; Hsp70; HIF1A; p53 |
| **Ke et al 2020 ^21^** | ARPE-19 | UC | R | 48 | X | X |  | X |  |  | ARPE-19; Rat Vitreous | CD63; CD81; CD9; Apaf1 |
| **Fukushima et al 2020 ^22^** | ARPE-19 | EQ | R | 72 | X | X |  | X | X |  | HUVEC | CD63; Hsp70; MCP-1; VEGF; IL-8; IGFBP-3; TIMP-1; ACTIVIN A; CD26; MMP-9; SERPIN E1; F3; THBS1; PTX3; |
| **Morris et al 2020 ^23^** | mouse  RPE/choroid | EQ | N/A | 16 |  |  |  |  | X |  |  | Tsg101 |
| **Mao et al 2020 ^24^** | ARPE-19 | UC | R | 48 | X | X | X |  |  |  |  | CD63; Tsg101; Hsp70 |
| **Gu et al 2020 ^25^** | ARPE-19 | UC | R | 72 | X | X |  |  |  |  | HUVEC | CD63; CD9 |
| **Ahn et al 2020 ^26^** | ARPE-19 | UC | R | 48 | X | X | X | X |  |  |  | CD63; CD9; CD81; SNTA1 |

***ARPE-19****: Cell line derived from the adult human retinal pigment epithelium;* ***Alix****: ALG-2-interacting Protein X;* ***BEST1****: Bestrophin-1;* ***CD****: Cluster of differentiation;* ***CFH****: Complement Factor H;* ***C3****: Complement 3;* ***CRYAB****: Crystallin alpha b;* ***ENO1****: α-enolase;* ***EQ****: ExoQuick;* ***EZR****: Ezrin;* ***F****: Flask;* ***FACS****: Fluorescence-activated cell sorting;* ***flot-1****: Flotillin-1;* ***F3****:* *Coagulation factor III;* ***G****: Density gradient;* ***hfRPE****: Human fetal retinal pigment epithelium;* ***HIF1A****:* *Hypoxia inducible factor 1 subunit alpha;* ***hiPSC-RPE****: Human induced pluripotent stem cells derived retinal pigment epithelium;* ***Hsp****: heat shock protein;* ***HUVEC****:* *Human umbilical vein endothelial cells;* ***IGFBP-3****:* *Insulin-like growth factor-binding protein 3;* ***IL-8****: Interleukin 8;* ***IL-1β****: Interleukin 1 beta;* ***Inc.****: Incubation time;* ***LAMP2****: Lysosomal Associated Membrane Protein 2;* ***MCP-1****: Monocyte chemoattractant protein-1;* ***MMP-9****: Matrix metallopeptidase 9;* ***mRPE****: mouse RPE;* ***MYOC****: Myocilin;* ***N/A****: Not applicable, not available, or no answer;* ***PDGFRβ****: Beta-type platelet-derived growth factor receptor;* ***pCD****: procathepsin D;* ***Prot****.: Proteomics;* ***PSAP****: prosaposin;* ***PTX3****: Pentraxin 3;* ***pRPE****: porcine primary RPE-retinal pigment epithelium;* ***qPCR****: quantitative PCR;* ***R****: Regular plate;* ***RLBP1****: Retinaldehyde binding protein 1;* ***RPE-65****: Retinal pigment epithelium-specific 65 kDa protein;* ***SDCBP****: Syntenin-1;* ***SLC39A12****: Solute carrier family 39 member 12;* ***SNTA1****: syntenin-1;* ***SEMA4A****: Semaphorin-4A;* ***TEM****: Transmission electron microscopy; ;* ***THBS1****: Thrombospondin 1;* ***TRL3****: Toll-like receptor 3;* ***Tsg101****:* *Tumor susceptibility 101;* ***TW****: Transwell insert;* ***UC****: Ultracentrifugation;* ***VEGFR****: vascular endothelial growth factor;* ***WB****: Western blot;* ***WBCs****: White blood cells;* ***X****: Performed;* *****: not beads used; **^†^** : in situ;

**Table S6. List of antibodies used in this study.**

| **Antibody** | **Company** | **Species** | **Dilution Work** | **Assay** |
| --- | --- | --- | --- | --- |
| MITF | MS-771-P1, NeoMarkers | Mouse | 1:50 | IF |
| VSX2 | AB9016, Millipore | Sheep | 1:500 | IF |
| EZRIN | DSHB | Mouse | 1 :500 | IF |
| OTX2 | AB9566, Millipore | Rabbit | 1:500 | IF |
| PAX6 | PAX6-s, DSHB | Mouse | 1:50 | IF |
| CRALBP | ab15051, Abcam | Mouse | 1:500 | IF/WB |
| BEST1 | NB300-164, Novus Bio | Mouse | 1:150 | IF/WB |
| RPE65 | ab78036, Abcam | Mouse | 1:100 | IF/WB |
| PMEL17 | HMB45, Dako | Mouse | 1 :500 | IF |
| ZO-1 | 61-7300, Invitrogen | Rabbit | 1:500 | IF |
| OCT4 | 60093, Stem Cell Tech | Mouse | 1:500 | IF |
| NANOG | 3580S, Cell Signaling | Rabbit | 1:500 | IF |
| SSEA4 | 60062BT, Stem Cell Tech | Mouse | 1:500 | IF |
| (β)-tubulin | E7-s, DSHB | Mouse | 1:500 | IF/WB |
| PHALLOIDIN | A12379, Invitrogen | N/A | 1:40 | IF |
| Na^+^/K^+^ ATPasa | DSHB | Mouse | 1:50 | IF |
| RHODOPSIN | ab98887, Abcam | Mouse | 1:500 | IF |
| RHODOPSIN | NBP2-251159, Novus Bio | Mouse | 1:5000 | WB |
| TSG-101 | sc-7964, Santa Cruz | Mouse | 1:1000 | WB |
| Flotillin-1 | 610820, BD Bioscience | Mouse | 1:1000 | WB |
| CD63 | sc-365604, Santa Cruz | Mouse | 1:500/1:1000 | IF/WB |
| GM130 | 610822, BD Bioscience | Mouse | 1:1000 | WB |
| CD81 | NBP1-77039, Novus Bio | Novus Bio | 1:500/1:1000 | IF |
| CLU | 66109-1-Ig, Proteintech | Mouse | 1:500/1:1000 | IF/WB |
| VTN | 66398-1-Ig, Proteintech | Mouse | 1:500/1:1000 | IF/WB |
| C3 | A15408, Antibodies.com | Rabbit | 1:1000 | WB |
| VIN | 26520-1-AP, Proteintech | Rabbit | 1:500/1:1000 | IF/WB |
| ApoE | 66830-1-Ig, Proteintech | Mouse | 1:500/1:1000 | IF/WB |
| NIAD-4 | 18520, Caiman Chemical | N/A | 1:1000 | IF |
| Β-Amyloid (6E10) | 803004, BioLegend | Mouse | 1:500/1:1000 | IF/WB |
| Β-Amyloid (4G8) | 800712, BioLegend | Mouse | 1:500 | IF |
| SOD1 | NBP2-24915, Novus Bio | Rabbit | 1:1000 | WB |
| Hsp70 | NBP2-47427, Novus Bio | Rabbit | 1:1000 | WB |
| Hsp90 | NB100-1972, Novus Bio | Mouse | 1:1000 | WB |
| TXN | 14999-1-AP, Proteintech | Rabbit | 1:1000 | WB |
| IL-13 | ab106732 , Abcam | Rabbit | 1:1000 | WB |
| TGF-β | #3711, Cell Signaling | Rabbit | 1:1000 | WB |
| APP | MAB348, Millipore Sigma | Mouse | 1:500/1:1000 | IF/WB |

**Table S7. List of oligos used in this study.**

| **Gene** |  | **Primer Sequence 5' to 3'** | **Annealing Temp.** | **Reference** |
| --- | --- | --- | --- | --- |
| *MITF* | F | TTCACGAGCGTCCTGTATGCAGAT | 60 | Meyer et al. 2009^27^ |
|  | R | TTGCAAAGCAGGATCCATCAAGCC |  |  |
| *OTX2* | F | ACCTTGAACTCCACCTCT | 56 | Maruotti et al. 2013^28^ |
|  | R | GCTTCTCTTCTCTGACTCTCTTTG |  |  |
| *TYR* | F | ATTGGGACTGGCGGGATG | 56 | Maruotti et al. 2013^28^ |
|  | R | GCATAAAGACTGATGGCTGTTG |  |  |
| *PMEL17* | F | GTGGTCAGCACCCAGCTTAT | 52 | Carr et al. 2009^29^ |
|  | R | GAGGAGGGGGCTGTTCTCAC |  |  |
| *CRALBP* | F | GCTGCTGGAGAATGAGGAAAC | 56 | Maruotti et al. 2013^28^ |
|  | R | TGGCTGGTGGATGAAGTGG |  |  |
| *RPE65* | F | TGCGTATGGACTTGGCTT | 56 | Maruotti et al. 2013^28^ |
|  | R | TCCTGCTCCTGGGCTCACC |  |  |
| *LRAT* | F | AGGATGCCAACGCCAAGTT | 52 | Carr et al. 2009^29^ |
|  | R | CCGCTGGTGGTCTTCGTATG |  |  |
| *BEST1* | F | CAGTTCTTCTTCTATGTTG | 54 | Maruotti et al. 2013^28^ |
|  | R | AATCATCATCATCCTCTC |  |  |
| *EMMPRIN* | F | GGAAAGTCACAGGTCACACG | 56 | Buchholz et al. 2009^30^ |
|  | R | GCCAAGAGGTCAGAGTCGTC |  |  |
| *SRP72* | F | TCTGCCTCTACAAGTAACATCAT | 58 | Synnergren et al. 2007^31^ |
|  | R | CTCATCACCAGCCACCTT |  |  |
| *CREBBP* | F | GAGAGCAAGCAAACGGAGAG | 60 | Synnergren et al. 2007^31^ |
|  | R | AAGGGAGGCAAACAGGACA |  |  |
| *EZRIN* | F | GTTTTCCCCAGTTGTAATAGTGCC | 60 | Tokunou et al. 2000^32^ |
|  | R | TCCGTAATTCAATCAGTCCTGC |  |  |
| *SILVER* | F | GTTGATGGCTGTGGTCCTTG | 56 | Buchholz et al. 2009^30^ |
|  | R | CAGTGACTGCTGCTATGTGG |  |  |
| *TYRP1* | F | AATGGATATTGCCTGTGTTTGC | 56 | Buchholz et al. 2009^30^ |
|  | R | AGTGATGGCTGTGGTCTTG |  |  |

(Continued from Table S7)

| **Gene** |  | **Primer Sequence 5' to 3'** | **Annealing Temp.** | **Reference** |
| --- | --- | --- | --- | --- |
| *TYRP2* | F | CTCAGACCAACTTGGCTACAGCTA | 56 | Buchholz et al. 2009^30^ |
|  | R | CAGCACAAAAAGACCAACCAAA |  |  |
| *TRANSTHYRETIN* | F | GATGGGATTTCATGTAACCAAGAG | 56 | Buchholz et al. 2009^30^ |
|  | R | CTGCCTGGACTTCTAACATAGC |  |  |
| *PEDF* | *F* | *AGATCTCAGCTGCAAGATTGCCCA* | *60* | Singh et al. 2013^33^ |
|  | *R* | *ATGAATGAACTCGGAGGTGAGGCT* |  |  |
| *ZO-1* | *F* | *GCAGCAATAAAGCAGCGTTTC* | *58* | Buchholz et al. 2009^30^ |
|  | *R* | *TTAGGGATTTCATGTAACCAAGAG* |  |  |
| *CLAUDIN-3* | *F* | *TACGACCGCAAGGACTACG* | *56* | Buchholz et al. 2009^30^ |
|  | *R* | *TGGTGGTGGTGGTGTTGG* |  |  |
| *EGFP* | *F* | *AAGTTCATCTGCACCACCG* | *60* | Current study |
|  | *R* | *TCCAGCAGGACCATGTGATCGC* |  |  |
| *HPRT1* | F | CCCTGGCGTCGTGATTAGTG | 60 | Synnergren et al. 2007^31^ |
|  | R | CCTGACCAAGGAAAGCAAG |  |  |
| *HMBS1* | F | GGCTGTTGCTTGGACTTCTC | 60 | Synnergren et al. 2007^31^ |
|  | R | TGCTATCTGGGGAGTGATTACC |  |  |

**Supplementary movie 1.** Confocal three-dimensional volume rendering of D50 ipRPE-P2 monolayers showing the organization of the actin filaments (labeled with phalloidin) confirming the advanced degree of polarization achieved by the cells.

**Supplementary movie 2.** Confocal three-dimensional volume rendering of a single RPE cell from D50 ipRPE-P2 monolayers displaying apical localization of melanin granules stained with PMEL17.

**Supplementary movie 3.** Confocal three-dimensional volume rendering of D50 ipRPE-P2 monolayer labeled with phalloidin (green) revealing the internalization of photoreceptor outer segments immunolabeled for rhodopsin (red).

**Supplementary movie 4.** Confocal three-dimensional volume rendering of D50 ipRPE-P2 monolayer labeled with phalloidin (green) showing photoreceptor outer segments on the cell apical surface of the RPE cells, at the level of the RPE microvilli (evidenced by punctate actin filaments) before being internalized.

**Supplementary movie 5.** CD81 showed cytoplasmic staining and prominent expression in punctate structures at the apical surface of D50 ipRPE-P2 monolayers.

**Supplementary movie 6.** Brownian motion exhibited by ipRPE-derived extracellular vesicles.

**References.**

1 Sonoda, S. *et al.* A protocol for the culture and differentiation of highly polarized human retinal pigment epithelial cells. *Nat Protoc* **4**, 662-673, doi:10.1038/nprot.2009.33 (2009).

2 McKechnie, N. M., Copland, D. & Braun, G. Hr44 secreted with exosomes: loss from ciliary epithelium in response to inflammation. *Invest Ophthalmol Vis Sci* **44**, 2650-2656 (2003).

3 Wang, A. L. *et al.* Autophagy and exosomes in the aged retinal pigment epithelium: possible relevance to drusen formation and age-related macular degeneration. *Plos One* **4**, e4160, doi:10.1371/journal.pone.0004160 (2009).

4 Sreekumar, P. G. *et al.* alpha B Crystallin Is Apically Secreted within Exosomes by Polarized Human Retinal Pigment Epithelium and Provides Neuroprotection to Adjacent Cells. *Plos One* **5**, doi:ARTN e12578

5 Gangalum, R. K., Atanasov, I. C., Zhou, Z. H. & Bhat, S. P. alpha B-Crystallin Is Found in Detergent-resistant Membrane Microdomains and Is Secreted via Exosomes from Human Retinal Pigment Epithelial Cells. *J Biol Chem* **286**, 3261-3269, doi:10.1074/jbc.M110.160135 (2011).

6 Toyofuku, T. *et al.* Endosomal sorting by Semaphorin 4A in retinal pigment epithelium supports photoreceptor survival. *Genes Dev* **26**, 816-829, doi:10.1101/gad.184481.111 (2012).

7 Biasutto, L., Chiechi, A., Couch, R., Liotta, L. A. & Espina, V. Retinal pigment epithelium (RPE) exosomes contain signaling phosphoproteins affected by oxidative stress. *Exp Cell Res* **319**, 2113-2123, doi:10.1016/j.yexcr.2013.05.005 (2013).

8 Locke, C. J. *et al.* Controlled exosome release from the retinal pigment epithelium in situ. *Exp Eye Res* **129**, 1-4, doi:10.1016/j.exer.2014.10.010 (2014).

9 Aboul Naga, S. H. *et al.* Intracellular pathways following uptake of bevacizumab in RPE cells. *Exp Eye Res* **131**, 29-41, doi:10.1016/j.exer.2014.12.010 (2015).

10 Singh, R. *et al.* Pharmacological Modulation of Photoreceptor Outer Segment Degradation in a Human iPS Cell Model of Inherited Macular Degeneration. *Mol Ther* **23**, 1700-1711, doi:10.1038/mt.2015.141 (2015).

11 Knickelbein, J. E. *et al.* Modulation of Immune Responses by Extracellular Vesicles From Retinal Pigment Epithelium. *Invest Ophth Vis Sci* **57**, 4101-4107, doi:10.1167/iovs.15-18353 (2016).

12 Gangalum, R. K., Bhat, A. M., Kohan, S. A. & Bhat, S. P. Inhibition of the Expression of the Small Heat Shock Protein alphaB-Crystallin Inhibits Exosome Secretion in Human Retinal Pigment Epithelial Cells in Culture. *J Biol Chem* **291**, 12930-12942, doi:10.1074/jbc.M115.698530 (2016).

13 Atienzar-Aroca, S. *et al.* Oxidative stress in retinal pigment epithelium cells increases exosome secretion and promotes angiogenesis in endothelial cells. *J Cell Mol Med* **20**, 1457-1466, doi:10.1111/jcmm.12834 (2016).

14 Klingeborn, M. *et al.* Directional Exosome Proteomes Reflect Polarity-Specific Functions in Retinal Pigmented Epithelium Monolayers. *Sci Rep* **7**, 4901, doi:10.1038/s41598-017-05102-9 (2017).

15 Shah, N. *et al.* Extracellular vesicle-mediated long-range communication in stressed retinal pigment epithelial cell monolayers. *Biochim Biophys Acta Mol Basis Dis* **1864**, 2610-2622, doi:10.1016/j.bbadis.2018.04.016 (2018).

16 Atienzar-Aroca, S. *et al.* Role of retinal pigment epithelium-derived exosomes and autophagy in new blood vessel formation. *J Cell Mol Med* **22**, 5244-5256, doi:10.1111/jcmm.13730 (2018).

17 Maisto, R. *et al.* ARPE-19-derived VEGF-containing exosomes promote neovascularization in HUVEC: the role of the melanocortin receptor 5. *Cell Cycle* **18**, 413-424, doi:10.1080/15384101.2019.1568745 (2019).

18 Oltra, M. *et al.* miR302a and 122 are deregulated in small extracellular vesicles from ARPE-19 cells cultured with H2O2. *Sci Rep* **9**, 17954, doi:10.1038/s41598-019-54373-x (2019).

19 Zhang, W. *et al.* Photo-Oxidative Blue-Light Stimulation in Retinal Pigment Epithelium Cells Promotes Exosome Secretion and Increases the Activity of the NLRP3 Inflammasome. *Curr Eye Res* **44**, 67-75, doi:10.1080/02713683.2018.1518458 (2019).

20 Ferreira, J. V. *et al.* Exosomes and STUB1/CHIP cooperate to maintain intracellular proteostasis. *Plos One* **14**, e0223790, doi:10.1371/journal.pone.0223790 (2019).

21 Ke, Y. *et al.* Exosomes derived from RPE cells under oxidative stress mediate inflammation and apoptosis of normal RPE cells through Apaf1/caspase-9 axis. *J Cell Biochem*, doi:10.1002/jcb.29713 (2020).

22 Fukushima, A., Takahashi, E., Saruwatari, J., Tanihara, H. & Inoue, T. The angiogenic effects of exosomes secreted from retinal pigment epithelial cells on endothelial cells. *Biochem Biophys Rep* **22**, 100760, doi:10.1016/j.bbrep.2020.100760 (2020).

23 Morris, D. R. *et al.* Exosomal MiRNA Transfer between Retinal Microglia and RPE. *Int J Mol Sci* **21**, doi:10.3390/ijms21103541 (2020).

24 Mao, K. & Wu, X. Microarray Analysis of Small Extracellular Vesicle-Derived miRNAs Involved in Oxidative Stress of RPE Cells. *Oxid Med Cell Longev* **2020**, 7658921, doi:10.1155/2020/7658921 (2020).

25 Gu, S. *et al.* Retinal pigment epithelial cells secrete miR-202-5p-containing exosomes to protect against proliferative diabetic retinopathy. *Exp Eye Res* **201**, 108271, doi:10.1016/j.exer.2020.108271 (2020).

26 Ahn, J. Y. *et al.* Release of extracellular vesicle miR-494-3p by ARPE-19 cells with impaired mitochondria. *Biochim Biophys Acta Gen Subj*, 129598, doi:10.1016/j.bbagen.2020.129598 (2020).

27 Meyer, J. S. *et al.* Modeling early retinal development with human embryonic and induced pluripotent stem cells. *Proc Natl Acad Sci U S A* **106**, 16698-16703, doi:10.1073/pnas.0905245106 (2009).

28 Maruotti, J. *et al.* A simple and scalable process for the differentiation of retinal pigment epithelium from human pluripotent stem cells. *Stem Cells Transl Med* **2**, 341-354, doi:10.5966/sctm.2012-0106 (2013).

29 Carr, A. J. *et al.* Protective effects of human iPS-derived retinal pigment epithelium cell transplantation in the retinal dystrophic rat. *Plos One* **4**, e8152, doi:10.1371/journal.pone.0008152 (2009).

30 Buchholz, D. E. *et al.* Derivation of functional retinal pigmented epithelium from induced pluripotent stem cells. *Stem Cells* **27**, 2427-2434, doi:10.1002/stem.189 (2009).

31 Synnergren, J. *et al.* Differentiating human embryonic stem cells express a unique housekeeping gene signature. *Stem Cells* **25**, 473-480, doi:10.1634/stemcells.2006-0247 (2007).

32 Tokunou, M. *et al.* Altered expression of the ERM proteins in lung adenocarcinoma. *Lab Invest* **80**, 1643-1650, doi:10.1038/labinvest.3780174 (2000).

33 Singh, R. *et al.* Functional analysis of serially expanded human iPS cell-derived RPE cultures. *Invest Ophthalmol Vis Sci* **54**, 6767-6778, doi:10.1167/iovs.13-11943 (2013).
