# Supplementary material for: Extracellular vesicles released by human retinal pigment epithelium mediate increased polarised secretion of drusen proteins in response to AMD stressors
Source: J Extracell Vesicles. 2021 Nov 8;10(13):e12165. doi: 10.1002/jev2.12165 (PMC8575963; doi:10.1002/jev2.12165)
Supplement: Supplementary file 10 — TableS4 [file JEV2-10-e12165-s010.docx]

**TABLE S4. Human stem cell-derived RPE systems.**

**Stepwise differentiation in adherent conditions**

| **Author** | **Cell type** | **Passage** | **Chamber** | **Coating** | **Cell density**  **(cells/cm^2^)** | **First RPE/**  **Characterization**  **(Days)** | **TER**  **(Ω cm^2^)** | **PH**  **assay** | **TEM** | **ICC** | **VEGF/PDEF** |
| --- | --- | --- | --- | --- | --- | --- | --- | --- | --- | --- | --- |
|  |  |  |  |  |  |  |  |  |  |  |  |
| Klimanskaya et al. 2004 ^1^ | hESC | 1-9 | R | GN | NA | 21-56/30 |  | X | X |  |  |
| Buchholz et al 2009 ^2^ | hiPSC/ hESC | 1-2 | R | GN | 6.3 x 10^4^ | 20-35/30 |  | X |  |  |  |
| Zahabi et al 2012 ^3^ | hiPSC/ hESC | 1 | R | MG | NA | 25/40-60 |  |  |  |  |  |
| Buchholz et al 2013 ^4^ | hiPSC/ hESC | 1 | TW/R | MG | NA | 14/30 |  | X |  |  |  |
| Maruotti et al. 2013 ^5^ | hiPSC/ hESC | 1-3 | TW/R | VN-PAS/MG | 1 x 10^5^ | 33-38/50 |  | X |  |  | X |
| Singh et al 2013 ^6^ | hiPSC | 1-3 | TW/R | LN | 1.4 x 10^5^ | 30/60 | X | X |  | X | X |
| Ferrer et al 2014 ^7^ | hiPSC/ hESC | 1-3 | TW/R | MG | 3.8 x 10^5^ | 25-35/42-56 | X |  | X | X |  |
| Reichman et al. 2014 ^8^ | hiPSC | 1-2 | R | MEF/GN | NA | 7/14 |  |  |  |  |  |
| Croze et al 2014 ^9^ | hiPSC/ hESC | 1-14 | TW/R | MG | 1 x 10^5^ | 28-42/45 |  | X |  |  | X |
| Leach et al 2015 ^10^ | hESC | 1-3 | TW/R | MG | NA | 14-32 |  | X |  |  | X |
| Maruotti et al. 2015 ^11^ **^*^** | hiPSC/ hESC | 1 | TW/R | VN-PAS/MG | 2.5-3 x 10^5^ | 28/35 |  | X |  |  | X |
| Lidgerwood et al 2016 ^12^ **^*^** | hiPSC/hESC | 1-2 | R | MG | 7.5 x 10^4^ | 20/60 |  | X |  |  |  |

**Differentiation from free-floating aggregates**

| **Author** | **Cell type** | **Passage** | **Chamber** | **Coating** | **Cell density**  **(cells/cm^2^)** | **First RPE/**  **Characterization** | **TER**  **(Ω cm^2^)** | **PH**  **assay** | **TEM** | **ICC** | **VEGF/PDEF** |  |
| --- | --- | --- | --- | --- | --- | --- | --- | --- | --- | --- | --- | --- |
|  |  |  |  |  |  |  |  |  |  |  |  |  |
| Klimanskaya et al. 2004 ^1^ | hESC | 1-9 | R | GN | NA | 28-56/30 |  | X | X |  |  |  |
| Osakada et al 2008 ^13^ | hESC | 1 | R | PDL/LN/FN | NA | 50/50-120 |  |  | X |  |  |  |
| Vugler et al. 2008 ^14^ | hESC | 1-2 | R | MG | 10 pigmented foci | 7-21/30 |  |  | X |  |  |  |
| Idelson et al. 2009 ^15^ | hESC | 1-2 | R | PDL/LN | 30-50 clusters | 28/21-35 |  | X | X |  |  |  |
| Vaajasaari et al. 2011 ^16^ | hiPSC/ hESC | 1 | TW/R | C-IV | NA | 10-21/28 | X | X |  |  | X **^†^** |  |
| Zhu et al. 2011 ^17^ | hESC | 1-6 | TW/R | GN/GX/FN | 2 x 10^5^ | 56/28 |  | X |  |  | X **^†^** |  |
| Wu et al. 2016 ^18^ | hESC | 1-2 | TW/R | MG | 1 x 10^5^ | 19/35-45 | X | X | X |  | X **^†^** | |
| Plaza-Reyes et al. 2016 ^19^ | hESC | 1 | TW/R | LN-521 | 0.6-1.2 x 10^4^ | 21/35 | X | X |  |  | X |  |
| Liu et al. 2018 ^20^ | hiPSC | 1-8 | TW | MG | NA | D26/60 | X |  | X | X | X | |

**Differentiation in retinal organoid cultures**

| **Author** | **Cell type** | **Passage** | **Chamber** | **Coating** | **Cell density**  **(cells/cm^2^)** | **First RPE/**  **Characterization** | **TER**  **(Ω cm^2^)** | **PH**  **assay** | **TEM** | **ICC** | **VEGF/PDEF** |
| --- | --- | --- | --- | --- | --- | --- | --- | --- | --- | --- | --- |
|  |  |  |  |  |  |  |  |  |  |  |  |
| Meyer et al. 2009 ^19^ | hiPSC/ hESC | NA | NA | LN | NA | 30-35/30 |  |  |  |  |  |
| Nakano et al. 2012 ^21^ | hESC | NA | NA | N/A | NA | 21/21 |  |  |  |  |  |
| Reichman et al. 2014 ^8^ | hiPSC | NA | NA | MEF | NA | 7/14 |  |  |  |  |  |
| Zhong et al. 2014 ^22^ | hiPSC | NA | NA | MG | NA | 16/16 |  |  |  |  |  |
| Wahlin et al. 2017 ^23^ | hiPSC/hESC | NA | NA | Non-coating | NA | 30-53/160 |  |  | X |  |  |

***This table summarizes the foundational protocols to generate human RPE tissue from stem cells; various modifications of these protocols have been used in more recent years. hESC****: human embryonic stem cells;* ***hiPSC****: human induced pluripotent stem cells;* ***R****: regular plate;* ***TW****: transwell insert;* ***GN****: gelatin;* ***MEF****: mouse embryonic fibroblasts;* ***MG****: Matrigel;* ***VN-PAS****: vitronectin peptide-acrylate surface;* ***PLD****: poly-D-lysine ;* ***FN****: fibronectin ;* ***LN****: laminin;* ***C-IV****: Collagenase-IV;* ***GX****: geltrex;* ***RPE****: retinal pigment epithelium;* ***TER****: transepithelial resistance;* ***PH****: phagocytosis;* ***TEM****: Transmission electron microscopy;* ***ICC****: intracellular calcium concentration: ;* ***VEGF****: Vascular endothelial growth factor;* ***PEDF****: Pigment epithelium-derived factor;* ***NA****: not applicable, not available, or no answer;* ***X****: performed;* ******* *One step approach: no selection or manual picking;* ***†*** *Only PEDF;*

1 Klimanskaya, I. *et al.* Derivation and comparative assessment of retinal pigment epithelium from human embryonic stem cells using transcriptomics. *Cloning Stem Cells* **6**, 217-245, doi:10.1089/clo.2004.6.217 (2004).

2 Buchholz, D. E. *et al.* Derivation of functional retinal pigmented epithelium from induced pluripotent stem cells. *Stem Cells* **27**, 2427-2434, doi:10.1002/stem.189 (2009).

3 Zahabi, A. *et al.* A new efficient protocol for directed differentiation of retinal pigmented epithelial cells from normal and retinal disease induced pluripotent stem cells. *Stem Cells Dev* **21**, 2262-2272, doi:10.1089/scd.2011.0599 (2012).

4 Buchholz, D. E. *et al.* Rapid and efficient directed differentiation of human pluripotent stem cells into retinal pigmented epithelium. *Stem Cells Transl Med* **2**, 384-393, doi:10.5966/sctm.2012-0163 (2013).

5 Maruotti, J. *et al.* A simple and scalable process for the differentiation of retinal pigment epithelium from human pluripotent stem cells. *Stem Cells Transl Med* **2**, 341-354, doi:10.5966/sctm.2012-0106 (2013).

6 Singh, R. *et al.* Functional analysis of serially expanded human iPS cell-derived RPE cultures. *Invest Ophthalmol Vis Sci* **54**, 6767-6778, doi:10.1167/iovs.13-11943 (2013).

7 Ferrer, M. *et al.* A multiplex high-throughput gene expression assay to simultaneously detect disease and functional markers in induced pluripotent stem cell-derived retinal pigment epithelium. *Stem Cells Transl Med* **3**, 911-922, doi:10.5966/sctm.2013-0192 (2014).

8 Reichman, S. *et al.* From confluent human iPS cells to self-forming neural retina and retinal pigmented epithelium. *P Natl Acad Sci USA* **111**, 8518-8523, doi:10.1073/pnas.1324212111 (2014).

9 Croze, R. H. *et al.* ROCK Inhibition Extends Passage of Pluripotent Stem Cell-Derived Retinal Pigmented Epithelium. *Stem Cells Transl Med* **3**, 1066-1078, doi:10.5966/sctm.2014-0079 (2014).

10 Leach, L. L., Buchholz, D. E., Nadar, V. P., Lowenstein, S. E. & Clegg, D. O. Canonical/beta-catenin Wnt pathway activation improves retinal pigmented epithelium derivation from human embryonic stem cells. *Invest Ophthalmol Vis Sci* **56**, 1002-1013, doi:10.1167/iovs.14-15835 (2015).

11 Maruotti, J. *et al.* Small-molecule-directed, efficient generation of retinal pigment epithelium from human pluripotent stem cells. *P Natl Acad Sci USA* **112**, 10950-10955, doi:10.1073/pnas.1422818112 (2015).

12 Lidgerwood, G. E. *et al.* Defined Medium Conditions for the Induction and Expansion of Human Pluripotent Stem Cell-Derived Retinal Pigment Epithelium. *Stem Cell Rev* **12**, 179-188, doi:10.1007/s12015-015-9636-2 (2016).

13 Osakada, F. *et al.* Toward the generation of rod and cone photoreceptors from mouse, monkey and human embryonic stem cells. *Nat Biotechnol* **26**, 215-224, doi:10.1038/nbt1384 (2008).

14 Vugler, A. *et al.* Elucidating the phenomenon of HESC-derived RPE: anatomy of cell genesis, expansion and retinal transplantation. *Exp Neurol* **214**, 347-361, doi:10.1016/j.expneurol.2008.09.007 (2008).

15 Idelson, M. *et al.* Directed Differentiation of Human Embryonic Stem Cells into Functional Retinal Pigment Epithelium Cells. *Cell Stem Cell* **5**, 396-408, doi:10.1016/j.stem.2009.07.002 (2009).

16 Vaajasaari, H. *et al.* Toward the defined and xeno-free differentiation of functional human pluripotent stem cell-derived retinal pigment epithelial cells. *Mol Vis* **17**, 558-575 (2011).

17 Zhu, D. *et al.* Polarized secretion of PEDF from human embryonic stem cell-derived RPE promotes retinal progenitor cell survival. *Invest Ophthalmol Vis Sci* **52**, 1573-1585, doi:10.1167/iovs.10-6413 (2011).

18. Wu, W. *et al.* Features specific to retinal pigment epithelium cells derived from three-dimensional human embryonic stem cell cultures - a new donor for cell therapy. *Oncotarget* **7**, 22819-22833, doi:10.18632/oncotarget.8185 (2016).

19 Plaza Reyes, A. *et al.* Xeno-Free and Defined Human Embryonic Stem Cell-Derived Retinal Pigment Epithelial Cells Functionally Integrate in a Large-Eyed Preclinical Model. *Stem Cell Reports* **6**, 9-17, doi:10.1016/j.stemcr.2015.11.008 (2016).

19 Meyer, J. S. *et al.* Modeling early retinal development with human embryonic and induced pluripotent stem cells. *Proc Natl Acad Sci U S A* **106**, 16698-16703, doi:10.1073/pnas.0905245106 (2009).

20 Liu, S. *et al.* Self-Formation of RPE Spheroids Facilitates Enrichment and Expansion of hiPSC-Derived RPE Generated on Retinal Organoid Induction Platform. *Invest Ophthalmol Vis Sci* **59**, 5659-5669, doi:10.1167/iovs.17-23613 (2018).

21 Nakano, T. *et al.* Self-formation of optic cups and storable stratified neural retina from human ESCs. *Cell Stem Cell* **10**, 771-785, doi:10.1016/j.stem.2012.05.009 (2012).

22 Zhong, X. *et al.* Generation of three-dimensional retinal tissue with functional photoreceptors from human iPSCs. *Nat Commun* **5**, 4047, doi:10.1038/ncomms5047 (2014).

23 Wahlin, K. J. *et al.* Photoreceptor Outer Segment-like Structures in Long-Term 3D Retinas from Human Pluripotent Stem Cells. *Sci Rep* **7**, 766, doi:10.1038/s41598-017-00774-9 (2017).
